# Reshaping tumor immune microenvironment through ROS-responsive prodrug polyplexes via synergistic effect of CRISPRi system and epigenetic inhibitor for breast cancer therapy

**DOI:** 10.1016/j.mtbio.2025.102285

**Published:** 2025-09-04

**Authors:** Huan Deng, Qianru Li, Bingxu Wang, Hong Yu, Shouzheng Sun, Zichen Li, Weizhen Pan, Qianfu Zhao, Heshuang Dai, Jiao Lu, Lihong Fan, Songwei Tan

**Affiliations:** a School of Chemistry, Chemical Engineering and Life Sciences, Wuhan University of Technology, Wuhan, 430070, China; b School of Pharmacy, Tongji Medical College, Huazhong University of Science and Technology, Wuhan, 430030, China; c Hubei Provincial Key Laboratory of Pediatric Genetic Metabolic and Endocrine Rare Diseases, Wuhan, 430030, China

**Keywords:** PD-L1, CRISPRi system, Epigenetic inhibitor, ROS-Responsive, Triple negative breast cancer

## Abstract

Engagement of programmed death-ligand 1 (PD-L1) on tumor cells with its receptor PD-1 on immune cells can transmit an inhibitory signal to induce immune evasion. Although the immune checkpoint inhibitor PD-L1 antibody has shown antitumor capability in clinical treatment, its wide clinical application still faces several side effects and individual selectivity. In our research, we utilized the Clustered Regularly Interspaced Short Palindromic Repeats interference (CRISPRi) system to suppress PD-L1 expression on breast cancer cells (4T1) and combined it with epigenetic inhibitor azacytidine (AZA) for enhanced cancer immunotherapy. Reactive oxygen species (ROS)-responsive poly(β-amino ester) (PBAE)-S-AZA cationic polymeric prodrug was fabricated, which could complex with CRISPRi plasmids to form the composite polyplexes via electrostatic interaction. The composite polyplexes could be taken up by tumor cells with high efficiency, followed by plasmid release with the cooperation of PBAE. The CRISPRi plasmids could lead to PD-L1 downregulation in tumor cells, leading to obvious relief of immune checkpoint blockade. In the meantime, the epigenetic inhibitor AZA was also released from the polyplexes due to the high intracellular ROS level, thereby enhancing the efficacy of immunotherapy via elevating MHC class I expression, enhancing antigen presentation, and inducing dendritic cell (DC) maturation. The ROS-responsive polyplexes helped to realize the combination of genome editing, immunotherapy, and epigenetic regulation. It will provide an effective platform for promoting antitumor treatment and precision medicine.

## Highlights

• Design of ROS-Responsive Polyplexes for Dual Cargo Delivery.
• Synergistic Effect of CRISPRi-Mediated PD-L1 Suppression and Epigenetic Reprogramming.
• Reshaping the Tumor Immune Microenvironment *in Vivo.*

## Graphical abstract

**Figure:**
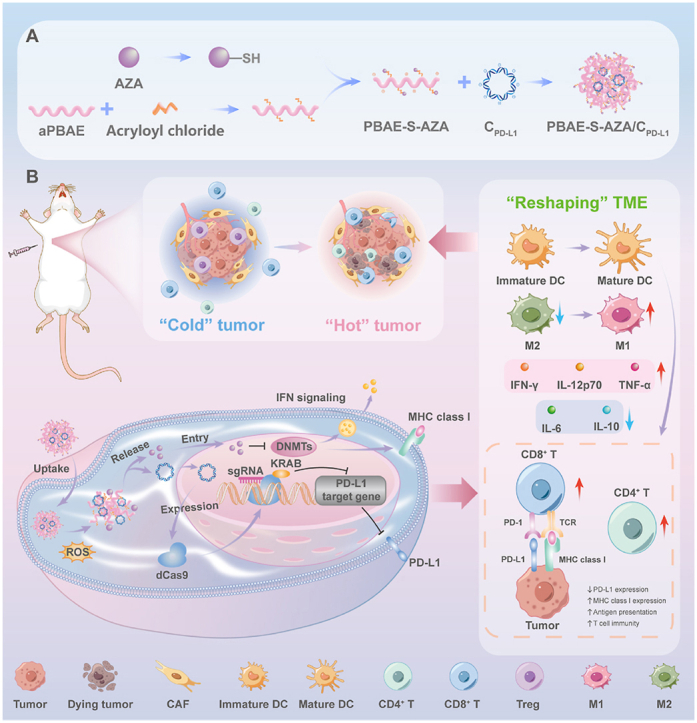

## 1 Introduction

The immunotherapy, especially immune checkpoint blockade (ICB), is considered a promising strategy for the treatment of various cancers [[1], [2], [3]]. Among the ICB inhibitors, the anti-programmed death-ligand 1(PD-L1) antibody has recently been explored as an innovative treatment against triple-negative breast cancer (TNBC). However, the preclinical trials indicated that it is only applicable to a small portion of TNBC patients. Several limitations, such as individual variability, immune evasion, and immune-related adverse events (irAEs), impeded its wide application [[4], [5], [6]].

High methylation of the antigen genes’ promoter induced reduction of tumor antigen expression is an important mechanism for immune therapy failure, which acts as a "molecular switch" for tumor immune escape, making it difficult for antigen-presenting cells to recognize the tumor antigen. Thus, small molecule inhibitors of DNA methyltransferase (DNMT) are the most widely used epigenetic therapies in antitumor treatment. Researches have revealed that DNMT inhibitors could not only increase genes expression related to antigen presentation, promote the presentation of neoantigens and induce the enhancement of major histocompatibility complex (MHC) class I molecules on tumor cells [7], but also elevate the expression of immune co-stimulatory molecules such as CD80, CD86 and CD40, thereby enhance the cytotoxicity of effector T cells on tumors [8]. Moreover, DNA demethylating drugs can induce an innate immune response in tumor cells by upregulating interferon (IFN)-α and IFN-β. The autocrine and paracrine signals of IFN-α/β in the tumor microenvironment can promote the production of pro-inflammatory cytokines and chemokines, thereby enhancing the immunogenicity of tumor cells [9]. However, DNMT inhibitors alone have low response in tumor treatment. On the contrary, as an "enhancer" of immunotherapy, they could significantly enhance the killing effect of immune cells [10,11]. In a phase II trial (NCT01928576) for non-small-cell carcinoma treatment, the combination of azacytidine (AZA, a nucleoside analog of DNMT inhibitors, which can effectively bind to DNA to form covalent complexes and promote the degradation of DNMT [12]) and nivolumab (anti-PD-1) showed that among the six patients who received AZA as pretreatment, five achieved 6-month progression-free survival after subsequent anti-PD-1, while the response rate for AZA was only 4 % [13,14].

IrAEs are another obstacle in anti-PD-1/PD-L1 antibody based immunotherapy. To solve these problems, recent researches proposed that modulating PD-L1 expression using the clustered regularly interspaced short palindromic repeat (CRISPR)-Cas9 system or siRNA could overcome immune evasion and reduce irAEs to some extent [[15], [16], [17], [18], [19]]. Thus, the gene therapy mediated ICB can be utilized as an alternative strategy to PD-L1 antibody therapy. With the optimization of gene editing tools, new systems which aim to activate or repress the transcription of target genes while minimizing off-target effects are being investigated [20,21]. The catalytically inactive "deactivated" Cas9 (dCas9), fused with the Krüppel-associated box (KRAB) transcriptional repression domain, which lacks the capability to cleave DNA, is widely adopted as a genetic engineering tool called the CRISPR interference (CRISPRi) system [22,23]. The CRISPRi system can effectively suppress the expression of the target locus without altering the genome, thus avoiding the accidental removal of functional regulatory elements [24]. Compared to the CRISPR system, the CRISPRi system can enhance the safety of genome editing in clinical applications by utilizing the dCas9, reducing the damage associated with off-target effects [25]. In contrast to siRNA technology, the CRISPRi system exhibits longer activity and superior efficiency in gene silencing [26]. Based on these findings, it can be inferred that the CRISPRi system can serve as a precise genetic regulatory tool for downregulating PD-L1 expression in TNBC. To further enhance the immunotherapeutic effect, we proposed a synergistic therapeutic strategy by combining gene editing technology with AZA treatment to enhance antigen presentation and reshape the tumor microenvironment.

**Scheme 1:**
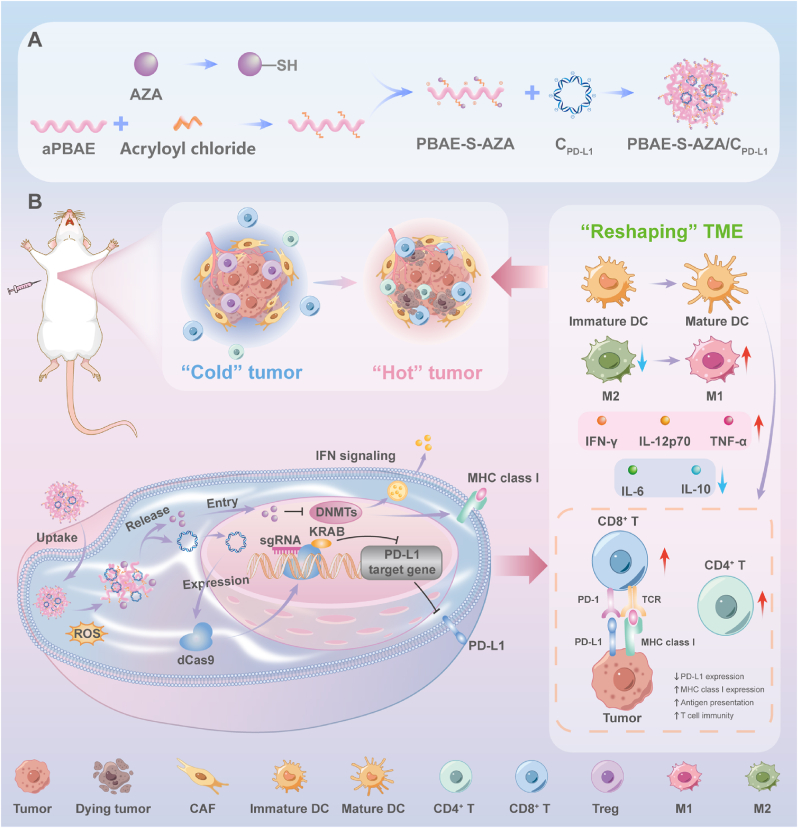
Schematic illustration of (A) ROS-responsive polyplexes for CRISPRi plasmid and AZA co-delivery and (B) ROS-responsive polyplexes mediated PD-L1 attenuation and tumor microenvironment remodulation.

Herein, we designed a reactive oxygen species (ROS)-responsive polyplex system for the co-delivery of PD-L1 specific targeted CRISPR/dCas9-KRAB plasmid (C_PD-L1_) and epigenetic inhibitor AZA. Firstly, based on our previous work [[27], [28], [29]], the ROS-responsive poly(β-amino ester) (PBAE)-S-AZA cationic polymer prodrug, coupled with a thioether bond, was designed and synthesized and served as the vehicle of gene cargo. Then, the optimized CRISPR/dCas9-KRAB plasmid, which could target PD-L1 with high efficiency, was combined with PBAE-S-AZA to form the composite polyplexes. After administration, the composite polyplexes could effectively penetrate tumor tissues and efficiently co-deliver the cargoes into tumor cells. The thioether bonds in the side chain were oxidized and rapidly hydrolyzed under high intracellular ROS conditions, releasing the plasmids and regulating intracellular ROS levels to some extent, thereby further improving the transfection efficiency. The plasmid was transcribed and translated to form dCas9-KRAB protein and single-guide RNA (sgRNA), specifically targeting and downregulating the expression of the target gene PD-L1 to relieve immune checkpoint inhibition and increase T cell activity. While the DNMT inhibitor AZA was simultaneously released to enhance the antitumor efficacy by elevating MHC class I expression, enhancing antigen presentation, and inducing dendritic cell (DC) maturation (Scheme 1). Therefore, the ROS-responsive polyplexes could achieve efficient co-delivery of the CRISPRi system and epigenetic inhibitor AZA, and lead to conversion of “cold” tumor to “hot” tumor via their synergistic effect.

In contrast to previous researches, the combination therapy may show several novelties as follows: Firstly, the CRISPRi system is more suitable for studies requiring fine-tuned gene expression. CRISPRi achieves gene silencing through dCas9 fused to transcriptional repressors KRAB, and its efficacy depends on high levels of dCas9 and sgRNA expression [30]. Therefore, effective inhibition can only be achieved in specific cell type driven by a strong promoter, whereas low levels of expression are not sufficient to have significant effects, thus reducing the disruption of non-target cells [31,32]. Secondly, the CRISPRi system only inhibits gene transcription without cutting DNA, making it safer than other genome editing tools. Thirdly, the ROS-responsive polyplex we fabricated could not only effectively co-deliver AZA and plasmids into tumor cells with precise ratiometric control, but also overcome side effects caused by poor biodistribution of free AZA *in vivo*, which further improved its efficacy in solid tumors [33]. Thus, our concept will provide new theoretical basis and therapeutic strategy for the precise targeting of TNBC with the combination of gene editing, immune regulation, and epigenetic therapy.

## 2 Materials and methods

### 2.1 Materials and reagents

pLvhU6-sgRNA hUbc-dCas9-KRAB-T2a-GFP plasmid was obtained from Addgene (#71237). pMax-GFP plasmid was purchased from Amaxa (Lonza, Basel, Switzerland). 5-Azacytidine (AZA) was purchased from Absin (China). The 5-amino-1-pentanol (AP) and 1,4-butanediol diacrylate (BDD) were purchased from TCI (Shanghai, China). The 1-(3-aminopropyl)-4-methylpiperazine (AMP) was from Adamas (Shanghai, China). Antibodies for western blot (WB) were purchased from Proteintech Group (USA) and Wuhan Servicebio Technology (China). Antibodies for flow cytometry (FCM) analysis were obtained from BD Biosciences (USA). Anti-mouse PD-L1 (B7-H1) antibody was purchased from Selleck (USA). Chlorin e6 (Ce6) was purchased from Macklin (Shanghai, China). Other chemical reagents were purchased from Aladdin (Shanghai, China).

### 2.2 Cell culture and animals

4T1 cells and B16F10 cells were purchased from the Cell Bank of Chinese Academy of Science (Shanghai, China). All the cells were cultured in Roswell Park Memorial Institute (RPMI) 1640 or Dulbecco's Modified Eagle Medium (DMEM), supplemented with 10 % heat-inactivated fetal bovine serum (FBS), 100 units/mL of penicillin, and 100 mg/mL of streptomycin at 37 °C, under 5 % CO_2_ and 95 % relative humidity atmosphere.

BALB/c mice (female, 18–20 g) were purchased from Hubei Biont Biological Technology Co., Ltd. All animals were fed and managed according to the guidelines of Laboratory Animals Ethics of HUST. The animal experiments were approved by the Institutional Animal Care and Use Committee at Tongji Medical College, HUST (IACUC Number:4060).

### 2.3 Synthesis and characterization of PBAE-S-AZA copolymer

AZA was reacted with acryloyl chloride (1:1.5, molar ratio) and 5 equiv of triethylamine in DMF at room temperature under pH 4–5 overnight. After filtering, this solution was slowly added dropwise to an aqueous solution containing 10 equiv of DTT in 10 vol (v/v) of water under stirring, followed by incubation at 4 °C overnight. The product was lyophilized to obtain raw product, and AZA-SH was purified by recrystallization in methanol.

PBAE was synthesized via Michael-type polymerization similar to our previous work [29]. In brief, BDD and AP (1:1, molar ratio) were accurately weighed and mixed. After stirring for 72 h at 90 °C, the resultant polymers were precipitated in cold diethyl ether and further washed to remove the unreacted monomers. Centrifuge the mixture at approximately 2000 rpm for about 3 min in a high-speed centrifuge. After vacuum drying, the polymers were reacted with acryloyl chloride to increase the content of acrylate groups. Finally, PBAE-S-AZA conjugate was obtained through the click reaction between the double bond and the thiol group on AZA-SH. The chemical structure of the product was characterized by ^1^H NMR spectra (Bruker AVANCE III 400 MHz NMR spectrometer, solvent: DMSO-*d*_6_) and Mass spectroscopy (microOTOF II, Bruker Daltonik, Germany).

### 2.4 Construction of CRISPRi system

The plasmid (pLvhU6-sgRNA hUbc-dCas9-KRAB-T2a-GFP) was cleaved with BsmbI restriction endonuclease to obtain linearized CRISPR/dCas9-KRAB plasmid. Multiple sgRNAs targeting the upstream promoter and 5′ UTR region of PD-L1 were designed and synthesized. These sgRNAs were then individually cloned into the linearized plasmid, followed by ligation to form a circular plasmid. The construction of the CRISPRi system was subsequently validated by sequencing.

### 2.5 Preparation and characterization of ROS-responsive polyplexes

The pMax-GFP plasmid was used as a model for characterization of the polyplexes. To prepare PBAE-S-AZA/pMax-GFP composite polyplexes, PBAE-S-AZA was dissolved in sodium acetate buffer (pH 5.0) with concentration of 50 μg/mL, and mixed with plasmid (1 μg/mL) at various mass ratios afterwards. The particle sizes and zeta potential of the PBAE-S-AZA/pMax-GFP were measured by dynamic light scattering (DLS, ZetaPALs, Brookhaven, USA). The stability of the complex polyplexes was evaluated in phosphate-buffered saline (PBS) with or without 10 % fetal bovine serum (FBS) at RT for 24 h. The morphology of the polyplexes was characterized by transmission electron microscopy (TEM, Tecnai G2-20, FEI, Netherlands). Furthermore, the drug release study was performed under different conditions (pH 5.0, pH 7.4, and pH 7.4 with 10 mM H_2_O_2_) at 37 °C. Polyplexes were loaded into dialysis (MVCO 500), and drug release was assessed at specified time points (1, 2, 3, 4, 6, 8, 12, 24, 36, and 48 h). Released AZA was quantified using HPLC (Shimadzu Corporation, Japan) with an Agilent 5 TC-C18 column (4.6 mm × 250 mm, 5 μm). The mobile phase consisted of potassium hydrogen phosphate (pH 6.5, 0.01 mol L^−1^) and methanol (90:10, v/v), with column temperature at 15 ± 2 °C and detected at 243 nm. Cumulative drug release percentages were calculated based on concentration measurements.

### 2.6 *In vitro* cytotoxicity assay

Briefly, 5 × 10^4^ cells were seeded into 96-well plates and cultured for 24 h. Then the culture medium was replaced and the cells were incubated with different concentrations of drugs (PBAE-S-AZA/pMax-GFP, PBAE/pMax-GFP, PBAE-S-AZA, PBAE, and AZA) for 4 h. Afterwards, the culture medium was replaced by fresh medium and cultured for another 20 h. Finally, the cell viability was evaluated by typical CCK-8 assay.

### 2.7 *In vitro* gene transfection assay

The cells were seeded in 6-well plates (2 × 10^5^ cells/well) 24 h before co-culture with PBAE-S-AZA/pMax-GFP. After incubating for 4 h, the culture medium was replaced with fresh medium and further incubated for 32 h, reaching the total transfection time of 36 h. The cells were observed under inverted fluorescence microscope (Olympus, Japan). The transfection efficiency was measured by flow cytometer (BD Accuri C6 plus, USA).

### 2.8 3D cell spheroid permeation assay

Agarose (20 mg) was fully dissolved by RPMI 1640 medium (1 mL) in the glass test tube, and then rapidly dispensed into 96-well plate once cooled to approximately 70 °C. The plate was then laid flat to cool for 4 h. The 4T1 cells were seeded into the 96-well plate with density of 300 cells/well and incubated for 24 h. When the 3D cell spheroids reached the diameter of approximately 500 μm, the drugs (PBAE-S-AZA/pMax-GFP, PBAE/pMax-GFP, and PEI/pMax-GFP) were added at mass ratios of 50:1, 50:1, and 4.8:1, respectively. After 36 h, the 3D cell spheroids were transferred into the glass-bottom confocal dish and then observed by laser scanning confocal microscopy (Sangon Biotech, China).

### 2.9 Cellular uptake studies

PBAE-S-AZA was labeled with Ce6 to prepare Ce6@PBAE-S-AZA/C_PD-L1_ polyplexes (C_PD-L1_ 1 μg/mL, Ce6@PBAE-S-AZA 50 μg/mL). After incubating with Ce6@PBAE-S-AZA/C_PD-L1_ polyplexes for 0.5, 1, 2, and 4 h, respectively, the cells were fixed and then stained with Hoechst 33258. The fluorescence of cells was observed by laser scanning confocal microscopy (Sangon Biotech, China). The transfection efficiency was measured by flow cytometer (BD Accuri C6 plus, USA).

The cells (2 × 10^5^ cells/well) were seeded in 6-well plates 24 h before being treated with different endocytosis inhibitors, including chlorpromazine hydrochloride (10 μg/mL), colchicine (2 μg/mL), and nystatin (50 μg/mL). Without removing the inhibitors, the cells were then incubated with Ce6@PBAE-S-AZA/C_PD-L1_ polyplexes (C_PD-L1_ 1 μg/mL, Ce6@PBAE-S-AZA 50 μg/mL) for 30 min. The cellular uptake efficiency was observed using flow cytometer (BD Accuri C6 plus, USA).

### 2.10 Cell membrane colocalization assays

The 4T1 cells were seeded into 12-well plates and cultured for 24 h. At various time points (0.5, 1, and 2 h), Ce6@PBAE-S-AZA/C_PD-L1_ polyplexes (PBAE 50 μg/mL, C_PD-L1_ 1 μg/mL) were added. After incubation and fixation, cells were stained with Dio Green (Beyotime, China) for 10 min at 37 °C, followed by Hoechst 33258 for 10 min at RT. Imaging was performed using laser scanning confocal microscopy (Sangon Biotech, China).

### 2.11 Lysosome escape study

The 4T1 cells were seeded into 12-well plates and cultured for 24 h. At various time points (1, 2, and 4 h), Ce6@PBAE-S-AZA/C_PD-L1_ polyplexes (PBAE 50 μg/mL, C_PD-L1_ 1 μg/mL) were added. Lyso-Tracker Green (Beyotime, China) stain was diluted in serum-free cell culture medium to achieve the final concentration of 50 nM. After incubation for specified periods, pre-warmed Lyso-Tracker Green working solution was added to the cells, and co-incubated for 1 h at 37 °C. After fixation, Hoechst 33258 staining solution was added and co-incubated with the cells for 10 min at RT. The cells were observed under laser scanning confocal microscopy (Sangon Biotech, China).

### 2.12 Intracellular ROS assay

The 4T1 cells were treated with PBAE-S-AZA/C_PD-L1_ polyplexes (C_PD-L1_ 1 μg/mL, Ce6@PBAE-S-AZA 50 μg/mL). The intracellular ROS level was detected using the fluorescent probe 2,7-dichlorodi hydro fluorescein diacetate (DCFH-DA) according to the instructions of the ROS detection kit (Beyotime, China). The fluorescence intensity of DCF was detected by inverted fluorescence microscopy (Olympus, Japan) after 0.5, 1, 2, 4, and 8 h, respectively. Moreover, the treated cells were collected and analyzed by flow cytometer (BD Accuri C6 plus, USA).

### 2.13 Generation and maturation of BMDCs

Bone marrow-derived dendritic cells (BMDCs) were isolated from the femurs and tibias of female BALB/c mice (6–8 weeks) and differentiated using GM-CSF (Abclonal, China). 4T1 cells were cultured in the upper chamber of the transwell overnight and treated with PBS, LPS (1 μg/mL), AZA (1 μM), PBAE (50 μg/mL), PBAE-S-AZA (50 μg/mL), PBAE/C_PD-L1_ (C_PD-L1_ 1 μg/mL, PBAE 50 μg/mL), or PBAE-S-AZA/C_PD-L1_ polyplexes (C_PD-L1_ 1 μg/mL, PBAE-S-AZA 50 μg/mL) subsequently. After 24 h, BMDCs were co-cultured in the lower chamber of transwell for an additional 24 h. Finally, BMDCs were harvested and analyzed by flow cytometry to detect the expression of CD11c, CD80, and CD86 to evaluate their maturation status.

### 2.14 RNA-seq transcriptomics

Transcriptome analysis was performed by Novogene (Beijing, China). Briefly, 4T1 cells were treated with or without PBAE-S-AZA/C_PD-L1_ polyplexes for 72 h. Then the total RNA was extracted and purified for further sequencing via an Illumina Novaseq platform. Gene Ontology (GO) and Kyoto Encyclopedia of Genes and Genomes (KEGG) pathway analyses for differentially expressed genes (DEGs) were conducted using the clusterProfiler R package. Analysis of gene sets of interest was performed using the Novomagic (https://magic.novogene.com).

### 2.15 Western blotting

The 4T1 cells were treated with AZA (1 μM), PBAE (50 μg/mL), PBAE-S-AZA (50 μg/mL), PBAE-S-AZA/pMax-GFP polyplexes (pMax-GFP 1 μg/mL, PBAE-S-AZA 50 μg/mL), and PBAE-S-AZA/C_PD-L1_ polyplexes (C_PD-L1_ 1 μg/mL, PBAE-S-AZA 50 μg/mL), respectively. The proteins were extracted and resolved on 10 % SDS-PAGE gel. Afterwards, the proteins were transferred to nitrocellulose membrane and incubated with corresponding primary antibodies at 4 °C overnight. Subsequently, the membranes were incubated with HRP-conjugated secondary antibodies for 1 h. The designated proteins were detected with enhanced chemiluminescence substrate (NCM Biotech, China) by GelView 6000 Pro Ⅱ (BLT, China).

### 2.16 *In vivo* biodistribution analysis

4T1 cells were injected in the right axillary of BALB/c mice subcutaneously. When the tumor volume reached 50–100 mm^3^, the mice were randomly divided into two groups, including the free Ce6 group and the Ce6@PBAE-S-AZA/C_PD-L1_. The Ce6@PBAE-S-AZA/C_PD-L1_ polyplexes (C_PD-L1_ 15 μg and Ce6@PBAE-S-AZA 750 μg per mouse) were paratumorally injected, and the mice were narcotized after 1, 3, 6, 12 and 24 h to observe the accumulation and retention of polyplexes in an imaging system (Pearl Imager, LICOR, USA). Finally, the mice were sacrificed after 24 h to collect the tumors, tumor-draining lymph nodes (TDLNs), and major organs (heart, liver, spleen, lung, and kidney) for analysis.

### 2.17 Antitumor effect *in vivo*

4T1 cells (2 × 10^6^ cells/per mouse) were subcutaneously inoculated in the right axillary of BALB/c mice. When the volume of tumors reached about 50–80 mm^3^, mice were randomly divided into 5 groups (n = 6) for various treatments, including PBS, AZA (3.66 μg per mouse), anti-PD-L1 Antibody (5 mg/kg), PBAE-S-AZA/pMax-GFP (GFP 15 μg and PBAE-S-AZA 750 μg per mouse) and PBAE-S-AZA/C_PD-L1_ (C_PD-L1_ 15 μg and PBAE-S-AZA 750 μg per mouse). The treatment started from the 6th day after inoculation and was repeated five times with an interval of three days. The tumor size and body weight of the mice were monitored every two days. The mice were defined as dead before the tumor size reached over 2000 mm^3^ and sacrificed when the mice became moribund. Survival rates were recorded for 60 days since tumor inoculation. Tumors and major organs (heart, liver, spleen, lung, and kidney) were fixed with 4 % paraformaldehyde for further analysis, including H&E, immunohistochemistry, and immunofluorescence staining sections.

### 2.18 Evaluation of antitumor immunity

To investigate the immune responses *in vivo*, another batch of mice was treated as aforementioned and sacrificed 24 days after inoculation. Tumors and TDLNs were collected for further experiments. To analyze the change in the tumor microenvironment, tumor tissues were digested, and the lymphocytes were subsequently purified. After being blocked by anti-CD16/32 antibody, the lymphocytes were stained with antibodies including FVS 510, BB700-CD3e, BV421-CD4, BB515-CD8a, APC-Cy7-CD45, APC-CD25, PE-Foxp3, PE-Cy7-CD11b, BV605-F4/80, BV711-CD206, and APC-R700-CD86 according to the protocol. To investigate the maturation of DC in TDLNs, the lymphocytes were purified and labeled with APC-CD11c, BV421-CD86, and PE-CD80 antibodies. All the lymphocytes were detected by flow cytometer (Sony ID7000™ Spectral Cell Analyzer) and analyzed using FlowJo software. The expression levels of cytokines, including interleukin-6 (IL-6), interleukin-10 (IL-10), interleukin-12p70 (IL-12p70), tumor necrosis factor-α (TNF-α), and interferon-γ (IFN-γ), were analyzed by ELISA kits (Absin, China).

### 2.19 Statistical analysis

All quantitative data, expressed as mean ± standard deviation (SD), were measured at least three times in parallel and analyzed using one-way ANOVA or Student's t-test. The statistical significance was set as a threshold of *p* < 0.05.

## 3 Results

### 3.1 Synthesis and characterization of PBAE-S-aza/plasmids polyplex

The ROS-responsive drug delivery system is a type of popular stimulus-responsive carrier that emerged in recent years. Compared to normal cells, tumor cells were demonstrated to produce higher levels of ROS, such as hydrogen peroxide and hydroxyl radicals, which could promote the breaking of linkages like thioethers, thioketals, selenium, and arylboronic esters, causing rapid drug release within tumor cells [34,35]. Therefore, ROS responsiveness was widely applied in drug delivery, including gene delivery [36,37]. Meanwhile, researches showed that exogenous cationic polymers could interact with mitochondria, leading to increase of ROS in cells and subsequently inducing oxidative stress. This kind of oxidative stress further affected the efficiency of gene transfection. Therefore, clearing excessive ROS produced in tumor cells and maintaining the normal intracellular physiological environment can enhance the gene transfection efficiency [38,39].

**Fig. 1:**
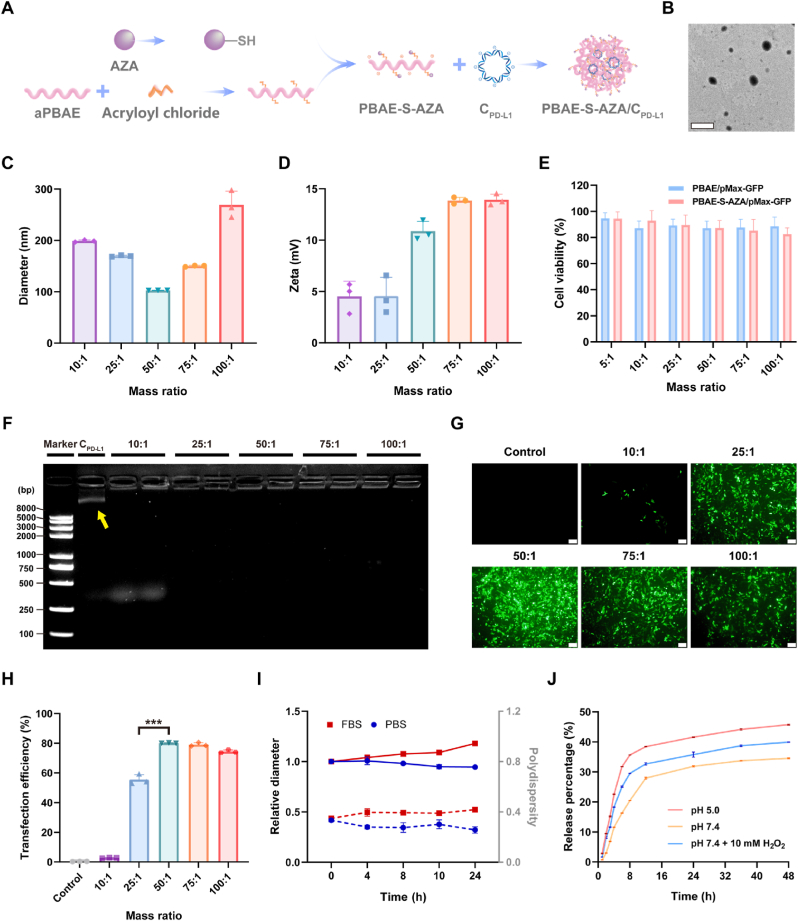
Synthesis and characterization of the ROS-responsive polyplexes. (A) Preparation diagram and (B) morphology of PBAE-S-AZA/pMax-GFP polyplexes. The scale bar is 500 nm. (C) Size distribution and (D) zeta potential of PBAE-S-AZA/pMax-GFP polyplexes with different weight ratios (n = 3). (E) Cell viability of PBAE/pMax-GFP and PBAE-S-AZA/pMax-GFP polyplexes with different weight ratios (n = 5). (F) Gel retardation assay of PBAE-S-AZA/pMax-GFP polyplexes with different weight ratios. (G) Fluorescence images of 4T1 cells that transfected with PBAE-S-AZA/pMax-GFP polyplexes. (H) Quantitative analysis of transfection efficiency in 4T1 cells that transfected with PBAE-S-AZA/pMax-GFP polyplexes. (I) Stability of PBAE-S-AZA/pMax-GFP polyplexes in PBS solution and FBS containing solution over 24 h (n = 3): relative diameter (solid line) and polydispersity (dashed line). (J) Release kinetics of AZA from PBAE-S-AZA/pMax-GFP polyplexes at pH 5.0, pH 7.4, or pH 7.4 with H_2_O_2_ buffers. The scale bar is 100 μm. Data were expressed as mean ± SD. ∗∗∗*p* < 0.001.

AZA is an insoluble drug (solubility <1 mg/ml). To achieve the precise delivery of AZA and plasmids, here, poly (β-amino ester) (PBAE) was further optimized via modifying the side hydroxyl groups on the side chain. By introducing ROS responsive thioether bonds, AZA was coupled with PBAE to form the cationic macromolecular prodrug PBAE-S-AZA, which was used to deliver plasmids (Fig. 1A, Fig. S1). The chemical structure was confirmed by ^1^H NMR spectra (Fig. S2). The spectra revealed characteristic amino peaks at 7.52 ppm (g’) for 10.13039/100005456AZA and 7.36 ppm (g’) for AZA-10.13039/100024172SH, confirming the successful synthesis of AZA-10.13039/100024172SH, which was further supported by mass spectrometry (Fig. S3), where characteristic peaks at *m/z* 267.07 (AZA) and 491.19 (AZA-SH) were observed. Meanwhile, the intermediate aPBAE spectrum showed hydrogen peaks at 2.35 ppm (a) and 2.64 ppm (b) corresponding to -CH_2_CH_2_COO- formation, which resulted from the reaction between the acrylate bond in BDD and the amine group in AP, indicating their successful reaction. After treating aPBAE with acryloyl chloride, the resulting aPBAE = exhibited a 5-fold increase in double bond content. The acrylate bond peaks at 6.39 ppm (k), 6.02 ppm (k'), and 6.25 ppm (j) observed in aPBAE = became invisible in PBAE-S-AZA, demonstrating the successful reaction between PBAE and AZA-SH. Furthermore, the characteristic AZA peaks (4.70–5.70 ppm) were clearly present in both AZA-SH and the final product PBAE-S-AZA, providing additional evidence for the successful conjugation of AZA to PBAE.

The composite polyplexes were complexed via electrostatic interactions due to the opposite charge of PBAE-S-AZA and plasmid. Utilizing the GFP-expressing plasmid (pMax-GFP) as a model, a series of PBAE-S-AZA/pMax-GFP polyplexes with different mass ratios, including 10:1, 25:1, 50:1, 75:1, and 100:1, were prepared to investigate the optimized polyplexes. The PBAE-S-AZA/pMax-GFP polyplexes exhibited uniform nano-sized spherical morphology (Fig. 1B), and the hydrodynamic diameter ranged from 100 nm to 300 nm. When the mass ratio was 50:1, it presented the smallest diameter, which was 102.76 ± 0.08 nm, and the ζ-potential was 10.87 ± 0.95 mV at this ratio (Fig. 1C and D, Fig. S4, and Table S1). The agarose gel electrophoresis demonstrated that the plasmid could be completely condensed by PBAE-S-AZA when the mass ratio was 10:1, indicating the high encapsulation efficiency of PBAE-S-AZA polymers (Fig. 1F).

To evaluate the stability of the polyplexes, PBAE-S-AZA/pMax-GFP polyplexes were prepared at a mass ratio of 50:1. The polyplexes exhibited distinct stability profiles in PBS solution and FBS containing solution at RT for 24 h. The polyplexes in PBS solution maintained the diameter ratio and PDI. In contrast, the diameter slightly increased to 1.18 times the initial particle size in FBS containing solution, suggesting moderate serum protein absorption (Fig. 1I).

To investigate the release behavior of AZA from PBAE-S-AZA/pMax-GFP polyplexes *in vitro*, the polyplexes were incubated at pH 5.0, pH 7.4, or pH 7.4 with H_2_O_2_ (10 mM) buffers to evaluate the pH and ROS responsibility of PBAE-S-AZA. The release rate of AZA was faster at pH 5.0 compared to pH 7.5, as evidenced by the 35.65 % release of AZA at pH 5.0 within 8 h, which was higher than the 20.45 % release at pH 7.5, indicating an acid-accelerated hydrolysis of ester bonds. On the other hand, compared to the control without H_2_O_2_, accelerated AZA release was observed in the presence of H_2_O_2_ at pH 7.4. At 8 h, 29.51 % AZA was released, which was 1.4 times of control group, and at 48 h, 39.92 % and 34.54 % of AZA was released, respectively. (Fig. 1J). These results showed that PBAE-S-AZA polyplexes display ROS-responsive and ROS-responsive release behavior due to their cleavable thioether bonds, which enable oxidation-triggered drug release due to the increased ester hydrolysis rate. This mechanism provided inspiration for precise spatiotemporal drug release within tumor tissues.

### 3.2 Construction of CRISPRi system to attenuate PD-L1 expression

The CRISPR/dCas9-KRAB plasmid was used as the vector, which contains a human U6 promoter-driven single guide RNA (sgRNA) expression cassette and a human Ubiquitin C promoter-driven catalytically inactive Cas9 (dCas9) fused to a KRAB repressor domain and a T2A-GFP reporter. Several sgRNAs were designed to target the upstream promoter and 5′ UTR region of PD-L1 gene, and separately cloned into the CRISPR/dCas9-KRAB vector to generate the C_PD-L1_ plasmids (Table S2, Fig. S5A, Fig. S5B, and Fig. S6). Each sgRNA containing C_PD-L1_ plasmids was transfected into 4T1 cells for 72 h separately, and the expression level of PD-L1 was detected by several methods to screen out the plasmid with the highest targeting efficiency. The flow cytometry results showed that the C_PD-L1_ plasmid containing sgRNA3 downregulated target gene most efficiently, which could attenuate 49.6 % of PD-L1 expression (Fig. S5C and D). Furthermore, we detected the expression of PD-L1 by real-time quantitative PCR (qRT-PCR) and Western Blot, both results were consistent with the flow cytometry, which identified that the PD-L1 expression could be significantly decreased after being transfected by the C_PD-L1_ plasmid containing sgRNA3 (Fig. S5E and F). In addition, to exclude the influence of AZA on PD-L1 expression, 4T1 cells were treated with different concentrations of AZA, ranging from 250 nM to 5 mM. It showed that the AZA had negligible impact on PD-L1 expression (Fig. S7). Therefore, the ROS-responsive polyplexes PBAE-S-AZA/C_PD-L1_ (PAC) were fabricated for further research.

### 3.3 The PAC polyplexes could efficiently enter tumor cells *in vitro*

**Fig. 2:**
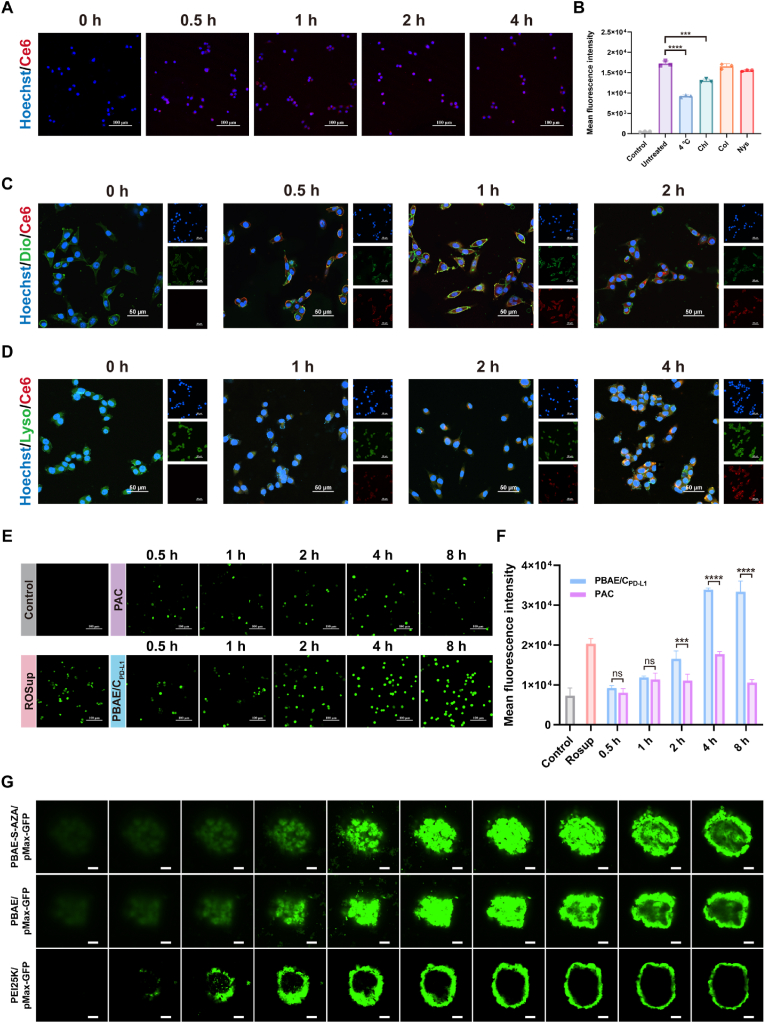
The PAC polyplexes could efficiently enter tumor cells *in vitro*. (A) Representative cellular uptake images of the PAC polyplexes at different time points. The scale bar is 100 μm. (B) Semi-quantitative analysis of 4T1 cells pretreated with endocytosis inhibitors or low-temperature (4 °C) followed by incubation with the PAC polyplexes (n = 3). (C) Representative fluorescence images of the interaction of polyplexes and 4T1 cell membrane at different time points. The scale bar is 50 μm. (D) Representative endo/lysosomal escape images of PAC polyplexes at different time points. The scale bar is 50 μm. (E) Representative fluorescence images of intracellular ROS levels in 4T1 cells that transfected with PAC polyplexes at different time points. The scale bar is 100 μm. (F) Quantitative analysis of intracellular ROS levels in 4T1 cells that transfected with PAC polyplexes (n = 3). (G) Fluorescence images of 3D cell spheroids that transfected with PBAE/pMax-GFP, PBAE-S-AZA/pMax-GFP and PEI 25K/pMax-GFP polyplexes. Data were expressed as mean ± SD. ∗∗∗*p* < 0.001, ∗∗∗∗*p* < 0.0001.

To investigate the cellular uptake of polyplexes, the conjugate PBAE-S-AZA was labeled with red fluorescent dye Ce6 and then complexed with PD-L1 targeting CRISPRi plasmid to form Ce6@PBAE-S-AZA/C_PD-L1_ (Ce6@PAC) polyplexes. As shown in Fig. 2A, the Ce6@PAC polyplexes can be rapidly internalized into 4T1 cells within 0.5 h and keep stable for at least 4 h (Fig. S8). To further elucidate the predominant endocytic pathways, we systematically investigated four key mechanisms as follows: (1) Suppress membrane fluidity via low-temperature incubation (4 °C), (2) Inhibit clathrin-mediated endocytosis with chlorpromazine hydrochloride, (3) Repress microtubule dynamics with colchicine, (4) Block caveolae-mediated pathways with nystatin. The results showed that there was a significant 46.32 % reduction of fluorescence intensity when the cells were pretreated with low temperature for 0.5 h (Fig. 2B). The reason might be that low temperature could decrease the fluidity of phospholipid membrane, thereby hampering membrane fusion and cellular internalization [40]. Conversely, chlorpromazine treatment resulted in a 24.48 % reduction in cellular uptake, demonstrating that clathrin-mediated endocytosis contributed partially to the internalization mechanism.

To further explore how the polyplexes entered 4T1 cells, we labeled the nuclei with Hoechst 33258 and the cell membrane with Dio Green. Observed by confocal laser scanning microscopy (CLSM), the yellow fluorescent in the images represented the overlap of membrane (green) and Ce6@PAC polyplexes (red) (Fig. 2C). After detecting at 0, 0.5, 1, 2 h, we found that the interaction of Ce6@PAC polyplexes and cell membrane was in time-dependent manner, which demonstrated that the Ce6@PAC polyplexes stated to confuse with cell membrane at 0.5 h and internalized into cell after 1 h. We proposed that the membrane fusion process was driven by electrostatic interactions between the positively charged polyplexes and the negatively charged cell membrane, leading to initial attachment and localized destabilization of the lipid bilayer, which facilitated direct polyplex internalization into the cytosol [41].

Endocytosis is also the pathway of cell uptake of PAC. After entry into cells, the successful endo/lysosomal escape of polyplexes is also vital for gene transfection. Therefore, the endo/lysosomal escape behavior of Ce6@PAC polyplexes was detected in 4T1 cells. As shown in Fig. 2D, the nuclei were stained by Hoechst 33258, and the endo/lysosome was labeled with Lysotracker Green. The yellow fluorescent in the images represented the overlap of endo/lysosome (green) and Ce6@PAC polyplexes (red), indicating the colocation of endo/lysosome and polyplexes. The endo/lysosomal tracking revealed that the transient colocalization of end/lysosome and Ce6@PAC polyplexes began at 1 h and decreased significantly by 4 h, indicating the separation of Ce6@PAC from endo/lysosome. These results, taken together, indicated that after cellular uptake, PAC is not trapped within endo/lysosomes, which creates favorable conditions for subsequent plasmid transfection.

To assess the capacity of thioether bonds in PBAE-S-AZA to decrease intracellular ROS, 4T1 cells were treated with PAC and PBAE/C_PD-L1_ polyplexes in the presence of fluorogenic ROS indicator DCFH-DA. Observed by fluorescent microscopy, the green fluorescence of the cells treated with PAC polyplexes was significantly weaker than that with PBAE/C_PD-L1_ polyplexes at all time points, including 2, 4, and 8 h, suggesting the thioether bonds in PBAE-S-AZA played an important role in scavenging intracellular ROS (Fig. 2E). Moreover, the level of ROS after being treated with PAC polyplexes exhibited an initial increase in 4 h, especially during the first hour (no significant difference from PBAE/C_PD-L1_) and followed by a decrease from 4 h to 8 h (Fig. 2F). The observed initial increase in intracellular ROS may be explained by the cellular stress response during polyplex internalization through the following mechanisms. First, the positively charged polyplexes may induce transient plasma membrane perturbations during cellular uptake, triggering NADPH oxidase (NOX) activation [42,43]. Secondly, the endosomal entrapment and subsequent escape of polyplexes can cause endosomal membrane disruption, leading to lysosomal leakage and the release of iron ions that catalyze Fenton reactions to produce ROS [44,45]. Thirdly, the internalized polyplexes may temporarily overwhelm cellular antioxidant systems (e.g., glutathione), creating an oxidative stress condition [46]. However, the subsequent decrease of ROS likely resulted from the oxidation of thioether bonds in PBAE-S-AZA, leading to the consumption of intracellular ROS. This dynamic behavior highlighted the ROS-scavenging capability of PBAE-S-AZA.

### 3.4 Transfection efficiency of PBAE-S-aza/plasmids polyplex

Before verifying the biocompatibility of PBAE polymer in 4T1 cells, we checked the cell viability by CCK8 assay (Fig. S9). The results showed that the cell viability was 94.83 % and 89.65 % when the cells were treated with PBAE at the concentration of 50 μg/mL for 24 h and 48 h, respectively. The cytotoxicity of AZA with different concentrations from 0.1 mM to 5 mM was also evaluated, and the cell viability of 4T1 cells was 89.49 % with the concentration of 1 mM after 48 h. PBAE-S-AZA also exhibited superior biosafety in 4T1 cells, which showed 88.39 % cell viability after 48 h at the concentration of 50 μg/mL. To further evaluate the safety of the composite polyplexes, we detected the cell viability of 4T1 cells treated with PBAE/pMax-GFP or PBAE-S-AZA/pMax-GFP with different mass ratios. It showed that the cell viability was over 80 % even when the mass ratio was 100:1 (Fig. 1E).

Furthermore, the transfection efficiency was assessed in 4T1 cell line using the pMax-GFP plasmid as model gene. At the mass ratio of 50:1, the composite polyplexes exhibited remarkable transfection efficiency of 80.10 % in 4T1 cells (Fig. 1G and H). These results demonstrated the high transfection capability of PBAE-S-AZA/pMax-GFP polyplexes in breast cancer cells. Based on these results, we could conclude that the most optimized mass ratio for the composite polyplexes was 50:1, which was chosen for the following experiments.

To explore the cellular permeability of composite polyplexes, we transfected PBAE-S-AZA/pMax-GFP into 3D cell spheroids, using PBAE/pMax-GFP and PEI 25K/pMax-GFP as controls. The results verified that the PBAE-S-AZA/pMax-GFP and PBAE/pMax-GFP polyplexes could effectively facilitate plasmid transfection in 4T1 cell lines and penetrate into spheroid structures, which was superior to PEI 25K/pMax-GFP (Fig. 2G).

Moreover, the cytotoxicity of PBAE, AZA, PBAE-S-AZA, and PBAE-S-AZA/pMax-GFP was examined in B16F10 cells, and it showed the same tendency as that in 4T1 cells with high safety (Fig. S10). The transfection efficiency of PBAE-S-AZA/pMax-GFP polyplexes depended on the mass ratio and varied by cell line. High efficiency was achieved in both B16F10 and N2a cells (Figs. S11 and S12), demonstrating the system's broad applicability for gene delivery.

### 3.5 The PAC polyplexes boosted immune response *in vitro*

**Fig. 3:**
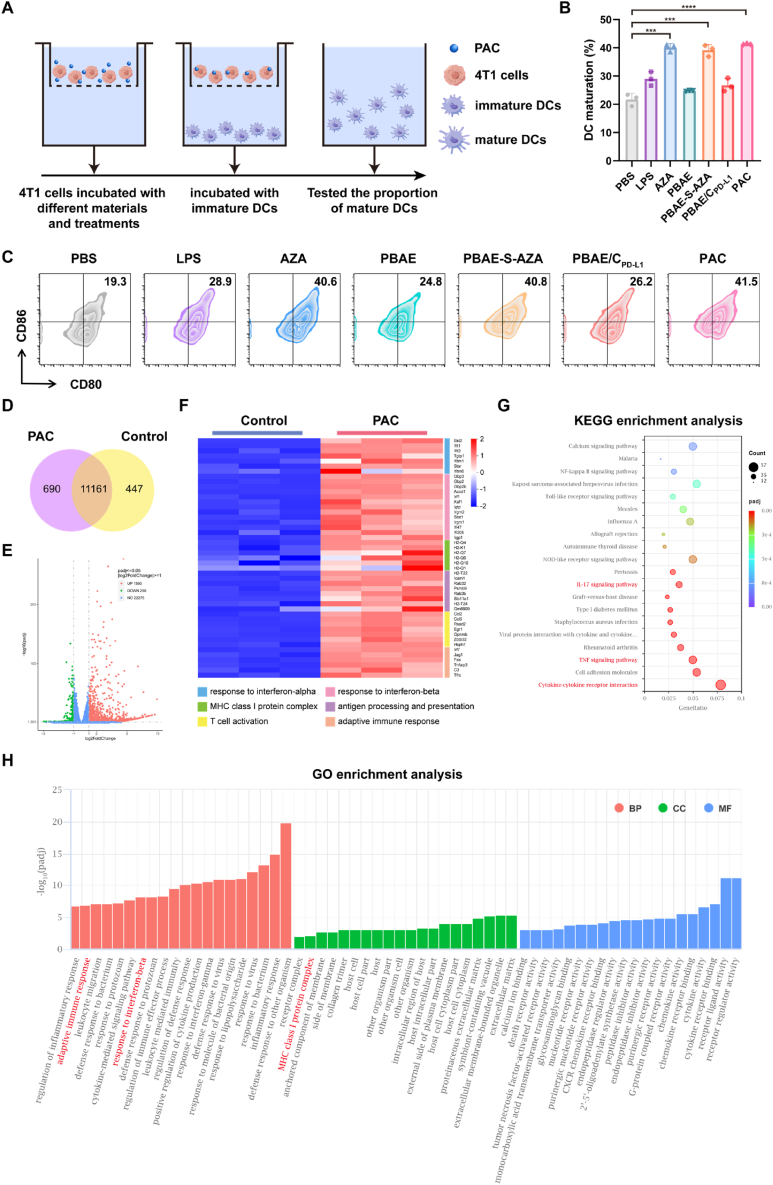
The PAC polyplexes boosted immune response *in vitro.* (A) Schematic illustration of transwell model for DCs maturation analysis. (B) Quantitative analysis and (C) representative flow cytometry plots of mature DCs after 24 h of various treatments (n = 3). (D) Venn diagram showing overlapped genes between PAC polyplexes and control group of 4T1 cells (n = 3). (E) Volcano plot showing differentially expressed genes (DEGs) between PAC group and control group (n = 3). (F) Heatmap showing significantly up-regulated and down-regulated genes of 4T1 cells receiving different treatments (fold change≥2 and *p* < 0.05) (n = 3). (G) Kyoto Encyclopedia of Genes and Genomes (KEGG) pathway enrichment analysis of DEGs (n = 3). The color represents the adjusted *p*-values, and the sizes of the spots represent the gene ratio (DEGs in pathway/total DEGs). (H) Gene Ontology (GO) enrichment analysis of DEGs in 4T1 cells, showing the top 20 significantly enriched terms in biological processes (BP), cellular components (CC), and molecular functions (MF) (n = 3). Data were expressed as mean ± SD. ∗∗∗*p* < 0.001, ∗∗∗∗*p* < 0.0001. (For interpretation of the references to color in this figure legend, the reader is referred to the Web version of this article.)

To investigate the impact of AZA on DC maturation, firstly, we isolated bone marrow-derived dendritic cells (BMDCs) from the tibia and femur of BALB/c mice. Then 4T1 cells were seeded into the upper chamber of transwell and treated with different drugs using PBS as negative control and LPS as positive control. Afterwards, the BMDCs were co-cultured in the lower chamber of transwell for 24 h and then collected to evaluate the maturation markers by flow cytometry (Fig. 3A). The expression of the maturation markers on the BMDCs, which were co-cultured with 4T1 cells pretreated with PBAE-S-AZA/C_PD-L1_ polyplexes obviously elevated, which showed that the population of CD80^+^CD86^+^ BMDC cells was 41.27 ± 0.21 %. It was 1.90 times compared to PBS group and 1.42 times to LPS group. The population of CD80^+^CD86^+^ BMDC cells in AZA group and PBAE-S-AZA group was similar to PBAE-S-AZA/C_PD-L1_ group, but significantly higher than PBAE group and PBAE/C_PD-L1_ group, indicating strong DC maturation elicited by AZA treatment (Fig. 3B and C, Fig. S13).

To elucidate the internal antitumor mechanism of polyplexes, we analyzed the transcriptomic profiles of 4T1 cells treated with PAC polyplexes. Transcriptomic analysis revealed 690 genes uniquely expressed in the PAC group and 447 genes specific to the control group (Fig. 3D). Compared to the control, 1880 differentially expressed genes (DEGs) were identified in the PAC group, including 1590 upregulated and 290 downregulated genes (Fig. 3E). A subset of these DEGs, implicated in antitumor immune regulation, was further analyzed (Fig. 3F). In 4T1 cells treated with PAC polyplexes, upregulation of genes associated with the IFN-α/β signaling pathway (Bst2, Ifit1, Ifitm1, Gbp3, Stat1), antigen presentation (H2-T22, Icam1, Rab32, Psmb9, H2-T24), and particularly MHC I-mediated processes (H2-Q4, H2-K1, H2-Q7, H2-Q6, H2-Q10) could be observed, which were consistent with previous researches [47,48]. In the meantime, the expression of PD-L1 was significantly downregulated on tumor cells after PAC treatment (Fig. S14A). It could be concluded that the combined immunostimulatory effects of PD-L1 knockdown and AZA could enhance T cell activation, leading to significant upregulation of adaptive immune response-related genes. These results suggested that PAC polyplexes could effectively improve antitumor efficacy.

To further explore the immune pathways activated by PAC polyplexes, Kyoto Encyclopedia of Genes and Genomes (KEGG) analysis was performed on the DEGs. This analysis highlighted cytokine-cytokine receptor interactions, including the IL-17 and TNF signaling pathways, as the key contributors to immune activation (Fig. 3G). Gene ontology (GO) enrichment analysis also supported these findings, demonstrating significant activation of immune-related pathways, such as adaptive immune response, response to IFN-β, and MHC I protein complex formation (Fig. 3H). Besides these, some immune-chemokine-related genes, such as Ccl2, Ccl5, Cxcl1, and Cxcl10 were significantly upregulated after treatment (Fig. S14B), which was similar to previous research [49]. These results provided valid evidence to prove that PAC polyplexes could enhance the antitumor effects by releasing the immune checkpoint blockade, enhancing antigen presentation, and increasing MHC I molecules on tumor cells at the transcriptomic level.

### 3.6 Distribution of PAC polyplexes *in vivo*

**Fig. 4:**
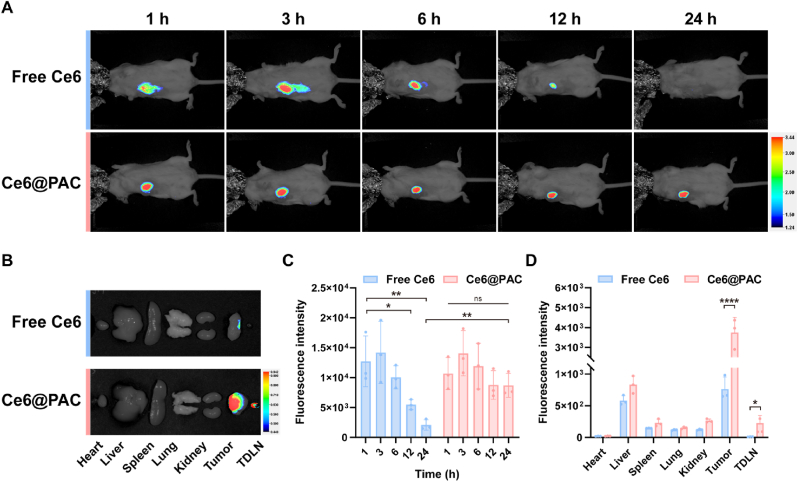
Distribution of PAC polyplexes *in vivo.* (A) *In vivo* fluorescence images of 4T1 tumor-bearing mice at 1, 3, 6, 12, and 24 h after paratumoral injection with free Ce6 and Ce6@PAC polyplexes (n = 3). (B) *Ex vivo* fluorescence images of tumor, TDLN, and major organs harvested from 4T1 tumor-bearing mice at 24 h after paratumoral injection with free Ce6 and Ce6@PAC polyplexes (n = 3). (C) Semi-quantitative analysis of tumor by *in vivo* imaging after paratumoral injection with free Ce6 and Ce6@PAC polyplexes at different time points (n = 3). (D) Semi-quantitative analysis of tumor, TDLN, and major organs harvested from 4T1 tumor-bearing mice at 24 h after paratumoral injection with free Ce6 and Ce6@PAC polyplexes (n = 3). Data were expressed as mean ± SD. ∗*p* < 0.05, ∗∗*p* < 0.01, ∗∗∗∗*p* < 0.0001.

To ascertain the tumor-retention of PAC *in vivo*, we injected Ce6@PAC and free Ce6 into 4T1 tumor-bearing mice paratumorally and tracked *in vivo* biodistribution using the IVIS imaging system. The results indicated that the fluorescence of free Ce6 diminished over time, and the signal almost disappeared at 24 h. In contrast, the Ce6@PAC group exhibited good retention in the tumor (Fig. 4A). *Ex vivo* distribution at 24 h confirmed favorable retention capabilities of PAC (Fig. 4B). To investigate the intratumoral distribution and retention of PAC polyplexes, *in vivo* fluorescence imaging was performed for semi-quantitative analysis at the injection site (Fig. 4C). The free Ce6 group exhibited rapid clearance from the injection site, with fluorescence intensity at 12 h declining to 43.04 % compared to 1 h (*p* < 0.05) and further diminishing to 16.43 % at 24 h (*p* < 0.01). In contrast, the Ce6@PAC polyplexes group demonstrated superior local retention, maintaining stable intratumoral fluorescence throughout the 24 h observation period, with no statistically significant difference between the 24 h and 1 h time points (*p* > 0.05). These results clearly indicated that the Ce6@PAC formulation significantly improved local drug retention compared to free Ce6. Additionally, semi-quantitative analysis of tissue fluorescence intensities revealed marked disparities at the tumor site between the Ce6@PAC and free Ce6 groups, while other principal organs showed negligible differences with minimal fluorescence (Fig. 4D). Meanwhile, fluorescence signals were found to obviously accumulate in tumor-draining lymph nodes (TDLNs), which was 13.4 times of free Ce6 (11.86) at 24 h *ex vivo*. This accumulation may be beneficial in promoting DC maturation and supporting antigen presentation, thereby enhancing the activation of anti-tumor immune responses.

### 3.7 The PAC polyplexes suppressed TNBC tumor growth

**Fig. 5:**
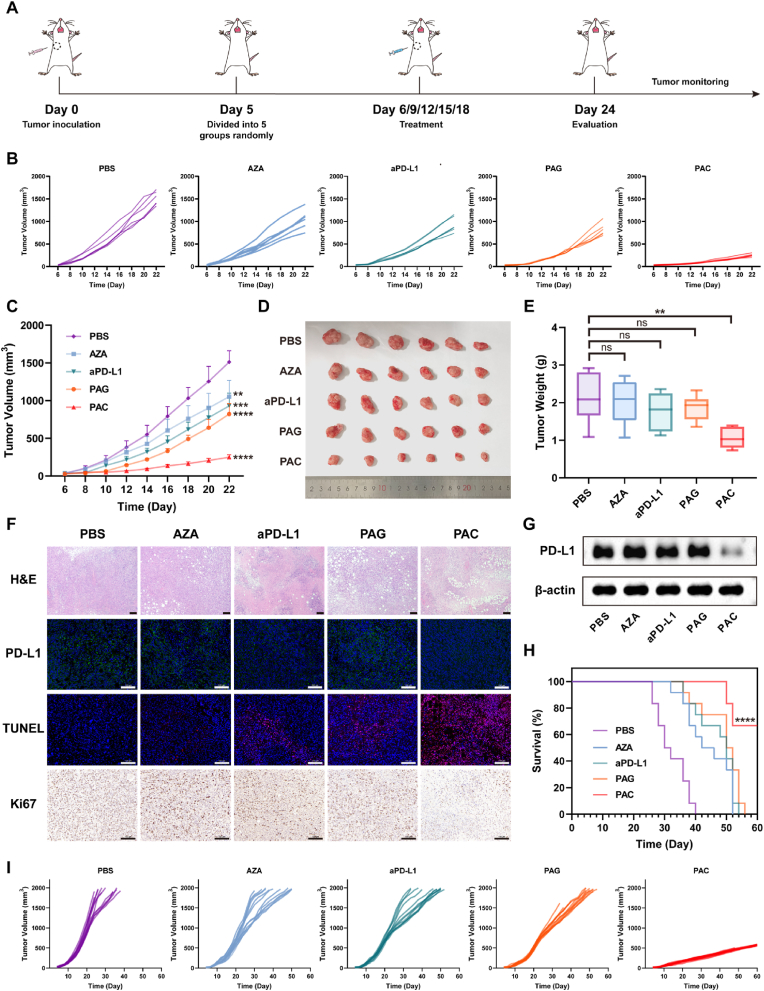
The PAC polyplexes suppressed TNBC tumor growth. (A) Treatment schedule of PAC mediated antitumor therapy. (B) Individual and (C) average tumor growth kinetics in 4T1 tumor-bearing mice receiving different treatments (n = 6). (D) Images of excised tumor tissues and (E) tumor weight for each treatment group (n = 6). (F) H&E, immunofluorescence, and immunohistochemistry staining analysis of PD-L1, TUNEL, and Ki67 in 4T1 tumor tissues after different treatments. The scale bar is 100 μm. (G) PD-L1 expression level in 4T1 tumor tissues receiving different treatments. (H) Survival time and (I) individual tumor growth kinetics of 4T1 tumor-bearing mice receiving different treatments (n = 12). Data were expressed as mean ± SD. ∗∗*p* < 0.01, ∗∗∗*p* < 0.001, ∗∗∗∗*p* < 0.0001.

Suppression of PD-L1 expression on tumor cells could weaken the interaction of PD-1/PD-L1, leading to restored T cell activation and enhanced antitumor responses. The epigenetic inhibitor, especially DNMTi, could not only increase the expression of tumor-associated antigens, but also increase MHC I expression, which ultimately mounted the visibility of tumor cells and DC cells to the adaptive immune system [47,50]. Therefore, we used CRISPRi system to downregulate PD-L1 expression in tumor cells and combined it with epigenetic inhibitor AZA for enhanced cancer immunotherapy. The therapeutic efficacy of the polyplexes PAC was investigated in 4T1 tumor-bearing mice by paratumoral injection (Fig. 5A). As shown in Fig. 5B and C, from the growth curve, it can be observed that the 4T1 tumors exhibited rapid growth in PBS group. The tumor growth could be slightly inhibited in AZA group, which might be attributed to induced DC maturation and increased immune activity boosted by AZA. The tumor volume of aPD-L1 group also decreased, similar to AZA group. We found that the tumor volume in the PAG group was smaller than that in the AZA group, possibly due to the enhanced retention capability of AZA by conjugated to PBAE. Importantly, the PAC group had extremely smallest tumor volume, with the size of only 200–300 mm^2^ on day 22, and the tumor inhibitory rate was 83.51 %, showing stronger antitumor efficacy compared to the aPD-L1 group. Furthermore, we dissected the tumors from the mice and calculated the tumor weight, which showed the similar tendency. The PAC group presented pronounced reduction, with the mean tumor weight of 1.060g, representing 50.30 % decrease relative to the PBS group (with the mean of 2.133 g) (Fig. 5D and E). These findings substantiated the PAC could enhance antitumor potency due to synergistic effect of PD-L1 suppression by CRISPRi system and AZA in the 4T1 tumor model.

Hematoxylin-eosin (H&E) staining images showed that the tumor tissue in PAC group was most significant. The immunofluorescent staining detected obvious PD-L1 downregulation in PAC group (Fig. 5F), indicating the high efficiency of CRISPRi system *in vivo*, which was also confirmed by Western blotting (Fig. 5G). Terminal deoxy-nucleotidyl transferase-mediated dUTP-biotin end labeling (TUNEL) also showed that the PAC group demonstrated the most pronounced tumor cell apoptosis and necrosis by TUNEL assay. Immunohistochemistry (IHC) analysis revealed diminished Ki67 and CD31 expression in the PAC group, implying retarded tumor growth rate (Fig. 5F, Fig. S15). Consistently, the results further validated the CRISPRi-mediated downregulation of PD-L1 within tumor tissues.

For the survival analysis, the PAC polyplexes prolonged the lifespan of 4T1 tumor-bearing mice, achieving 66.67 % survival rate by day 60 (Fig. 5H). This survival extension in the PAC group was attributed to the attenuated tumor growth rate, as illustrated by the long-term tumor growth trajectories (Fig. 5I). In addition, no obvious loss of body weight was detected in all groups throughout the treatment period, and histological examination of major organs via H&E staining confirmed the good biosafety of the PAC-based delivery system *in vivo* (Figs. S16, S17, S18). Serum biochemistry analysis revealed that key parameters, including alanine aminotransferase (ALT), aspartate transaminase (AST), and blood urea nitrogen (BUN), all remained within normal ranges across all treatment groups, which further supported biocompatibility of the system (Fig. S19).

### 3.8 The PAC polyplexes potentiated antitumor immune response *in vivo*

**Fig. 6:**
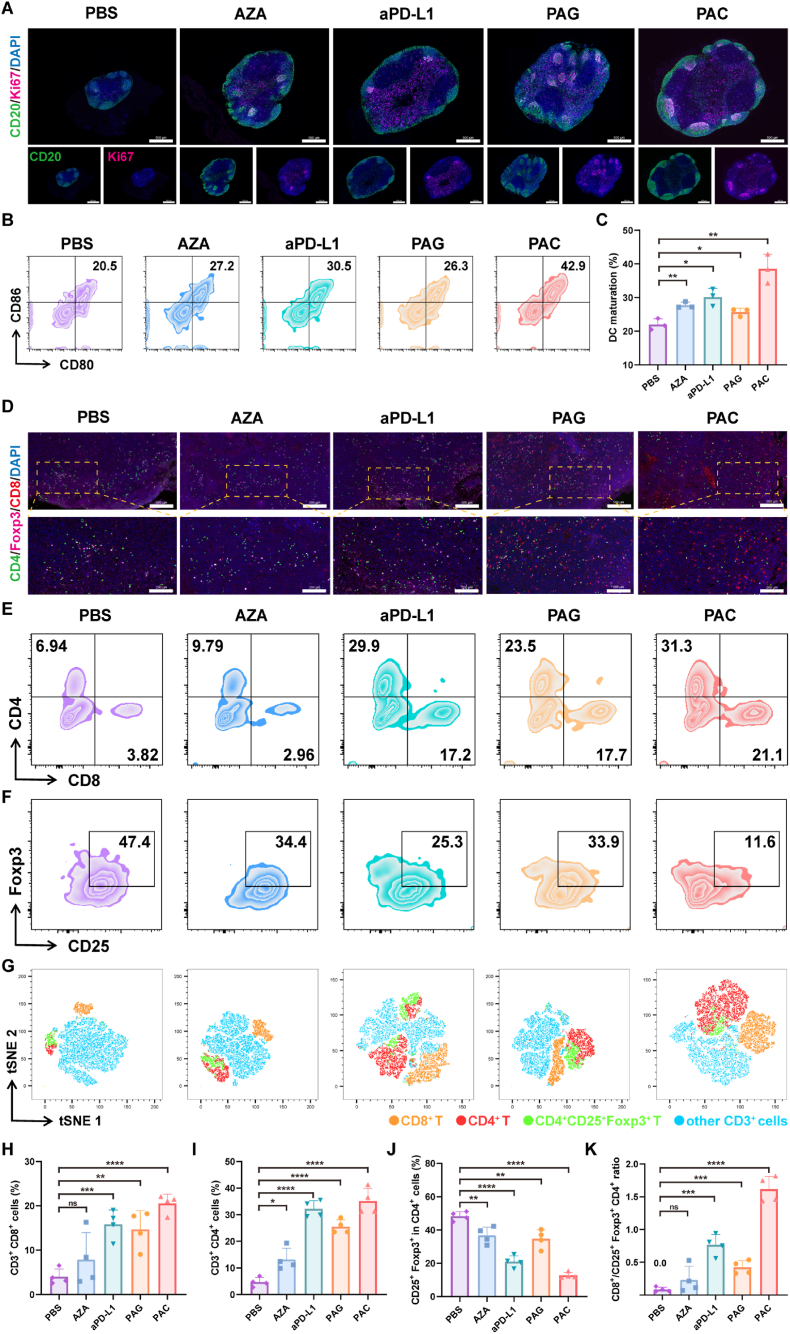
The PAC polyplexes potentiated antitumor immune response *in vivo.* (A) Representative immunofluorescence staining images of the proliferation of germinal centers of TDLNs in 4T1 tumor-bearing mice. The scale bar is 500 μm. (B) Representative flow cytometry plots and (C) quantitative analysis of mature DCs in TDLNs (n = 3). (D) Representative immunofluorescence staining images of tumor sections showed CD4^+^ T cells, CD8^+^ T cells, and Tregs infiltration in tumor microenvironment. The scale bars are 500 μm (upper panels) and 200 μm (lower panels). Representative flow cytometry plots of (E) CD8^+^ and CD4^+^ T cells, (F) Tregs (CD4^+^CD25^+^Foxp3^+^) in tumor microenvironment. (G) t-SNE map of immune cells in tumor microenvironment. Quantitative analysis of (H) CD8^+^ T cells, (I) CD4^+^ T cells, and (J) CD25^+^Foxp3^+^ T cells gating on CD4^+^ T cells in tumor microenvironment (n = 4). (K) Ratios of CD8^+^ T cells and Tregs (CD4^+^CD25^+^Foxp3^+^) in tumor microenvironment (n = 4). Data were expressed as mean ± SD. ∗*p* < 0.05, ∗∗*p* < 0.01, ∗∗∗*p* < 0.001, ∗∗∗∗*p* < 0.0001.

The mechanisms of PAC in boosting antitumor immune response were investigated by analyzing the TDLNs and tumor microenvironment. The lymphocytes in TDLNs are one of the keys to the initiation of immune response [51]. Therefore, the TDLNs were dissected for immunofluorescent staining. As shown in Fig. 6A, PAC-treated mice developed markedly enlarged TDLNs compared to PBS controls, with increased fluorescence intensity for both CD20 (a pan-B cell marker) and Ki67 (a proliferation marker). Importantly, PAC treatment promoted robust germinal center formation, as demonstrated by intense follicular fluorescence, indicating active B-cell proliferation and differentiation. These findings demonstrated that PAC polyplexes could effectively stimulate humoral immune responses.

Mature DCs are pivotal in initiating adaptive immunity, determining immune response outcomes, and maintaining the balance between tolerance and inflammation [52]. Therefore, the maturation of DCs in TDLNs was detected by flow cytometry. The results indicated that the percentage of mature DCs in PAC groups reached a maximum of 38.53 %, which increased by 16.53 % and 8.40 % to PBS (22.00 %) and aPD-L1 (30.13 %) groups, respectively (Fig. 6B and C, Fig. S20), indicating that the combination therapy was beneficial to induce DC maturation.

To further demonstrate that C_PD-L1_-AZA combination could boost anti-tumor immune response in tumor microenvironment, the population of tumor infiltration lymphocytes (TILs), including CD8^+^T cells, CD4^+^T cells, Tregs and macrophages in the tumor tissues of 4T1 tumor-bearing mice, was analyzed after treatment. The immunofluorescence staining in Fig. 6D showed that higher infiltration of CD8^+^T cells and CD4^+^T cells could be observed in the mice treated with PAC polyplexes than PBS, while the infiltration of Tregs decreased in PAC group. Tumor tissues were further digested to separate TILs for flow cytometry analysis (Fig. 6E and F, Fig. S21), and the main clusters were identified by t-SNE analysis (Fig. 6G). The results showed that the percentage of CD3^+^CD8^+^ cells increased to 20.53 %, compared to 4.02 % in the PBS group, 15.83 % in the aPD-L1 group, and 7.87 % in the AZA group, implying increased proportion of CTLs in tumors after treatment as expected (Fig. 6H). Similar results were obtained for intratumoural infiltration of CD4^+^ T cells, the percentage of CD3^+^CD4^+^ cells in tumor microenvironment significantly elevated to 35.10 % (Fig. 6I).

Meanwhile, Tregs (CD4^+^CD25^+^Foxp3^+^), which could induce immune tolerance and inhibit the activity of CD4^+^ and CD8^+^ T cells, are also essential for antitumor efficacy. As shown in Fig. 6J, the percentage of Tregs in PAC group was much lower than other groups. Importantly, the ratio of CTLs to Tregs reached 1.62 in the PAC group, compared to 0.08 in the PBS group and 0.76 in the aPD-L1 group (Fig. 6K).

**Fig. 7:**
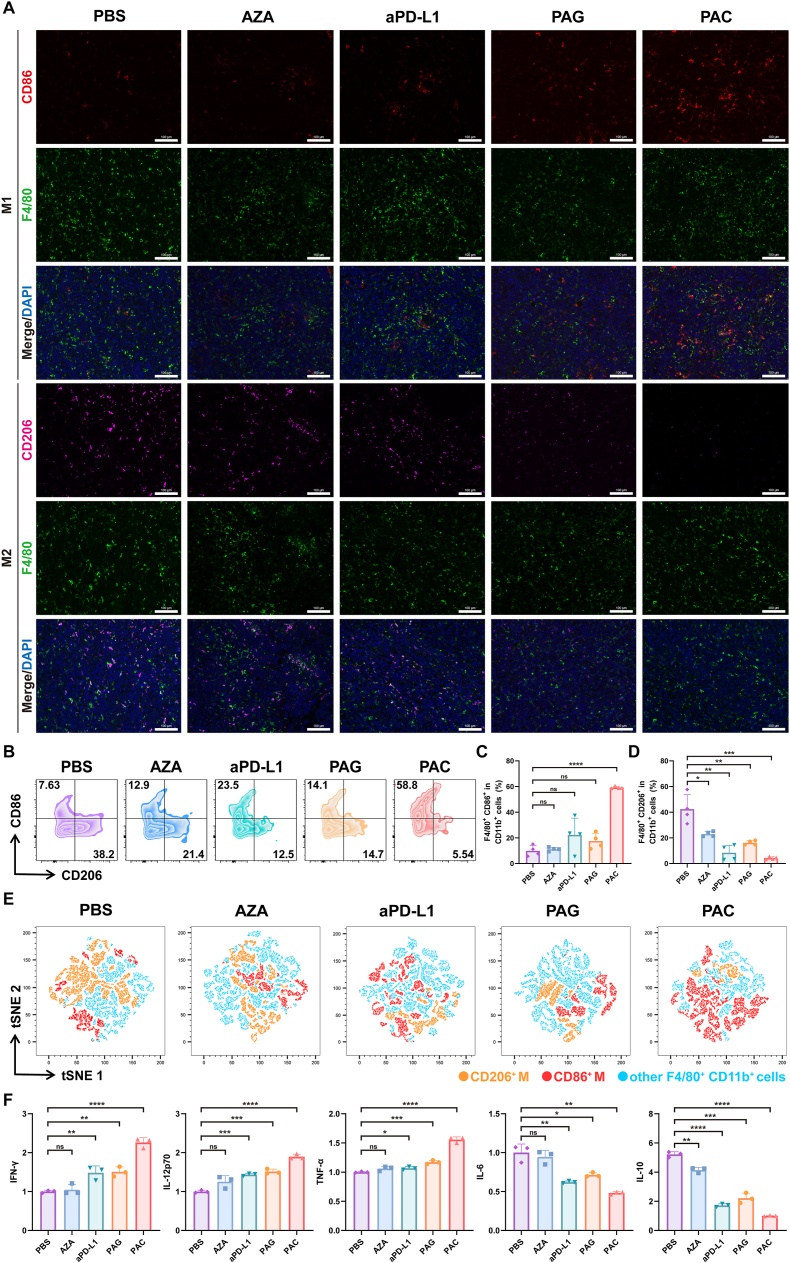
The PAC polyplexes potentiated antitumor immune response *in vivo.* (A) Representative immunofluorescence staining images of tumor sections showed M1-like macrophages (F4/80^+^CD86^+^) and M2-like macrophages (F4/80^+^CD206^+^) infiltration. The scale bar is 100 μm. (B) Representative flow cytometry plots of M1-like macrophages (CD11b^+^F4/80^+^CD86^+^) and M2-like macrophages (CD11b^+^F4/80^+^CD206^+^) in tumor. Quantitative analysis of (C) M1-like macrophages (F4/80^+^CD86^+^) and (D) M2-like macrophages (F4/80^+^CD206^+^) gating on CD11b^+^ cells in tumors (n = 4). (E) t-SNE map of immune cells in tumor microenvironment. (F) The levels of IFN-γ, IL-12p70, TNF-α, IL-6, and IL-10 in tumor microenvironment were analyzed (n = 3). Data were expressed as mean ± SD. ∗*p* < 0.05, ∗∗*p* < 0.01, ∗∗∗*p* < 0.001, ∗∗∗∗*p* < 0.0001.

The M2 cells (CD11b^+^F4/80^+^CD206^+^) in macrophages are another important portion of immunosuppressive cells. In our research, we found that there was obvious increase of M1 cells (CD11b^+^F4/80^+^CD86^+^) and decrease of M2 cells in PAC group detected by immunofluorescence images (Fig. 7A, Fig. S22). To quantify the percentage of macrophages in tumor microenvironment, flow cytometry analysis was performed (Fig. 7B). As shown in Fig. 7C, the percentage of M1 cells in PAC group increased to 59.23 %, compared to 9.90 % in the PBS group and 22.29 % in the aPD-L1 group. In contrast, the percentage of M2 cells in PAC group decreased to 4.31 %, which was 42.43 % in PBS group (Fig. 7D). We also conducted t-SNE dimension analysis to identify the main clusters in tumor microenvironment (Fig. 7E). These results suggested that the PAC polyplexes could not only effectively enhance antitumor immunity be elevating CTLs but also modulate tumor suppressive microenvironment through reducing Tregs and polarizing M2 to M1. In addition, the levels of cytokines IFN-γ, IL-12p70, TNF-α, IL-6, and IL-10 in tumors were measured after various treatments. Remarkably, C_PD-L1_ plus AZA combination therapy could significantly upregulate the levels of IFN-γ, IL-12p70, TNF-α, and downregulate the levels of IL-6, IL-10 in tumor tissues (Fig. 7F).

The systematic comparison of five treatment groups revealed the unique advantages of PAC combination therapy. First, to examine the adjuvant effects of the PBAE carrier and the immunomodulatory role of thioether bond modification, the AZA monotherapy group served as the control. The AZA group showed certain T-cell activation through epigenetic regulation, with the CD3^+^CD8^+^ cells proportion reaching 7.87 %, performing only slightly better than the PBS group. This highlights the limitations of epigenetic regulation alone. On the other hand, the PAG group showed significantly better results than the AZA group, proving that the PBAE carrier, with its effective tumor targeting, tissue retention, and responsive release properties, can effectively enhance AZA's immune-activating effects. The PAC group (containing the PD-L1 knockdown plasmid) not only outperformed the PAG group but also did better than the aPD-L1 monoclonal antibody group, demonstrating successful PD-L1 knockdown using the CRISPRi system. Overall evaluation showed an efficacy ranking: PAC > aPD-L1> PAG > AZA ≈ PBS. These findings highlight the power of combining epigenetic regulation and immune checkpoint inhibition through the responsive-release PBAE system. PAC therapy's dual action—enhancing tumor immunogenicity while blocking immune escape mechanisms—offers a promising approach for treating “cold” tumors that resist conventional therapies.

## 4 Discussion

In recent years, the ICB that targets PD-1/PD-L1 axis (e.g., perbrolizumab) has been approved as an ideal immunotherapy for breast cancer, while it is prone to benefit the patients marked by high TILs infiltration and immune checkpoint expression in tumor tissues [53]. Therefore, it highlights the importance of developing combination strategies in order to convert the tumor microenvironment and improve the ICB efficacy in treating TNBC, which is a highly immune-suppressive subtype of breast cancer. Epigenetic regulation is now acknowledged as a key hallmark of cancer and explored to augment therapeutic responses to various treatments, including chemotherapy and immunotherapy [54]. Among these epigenetic drugs, the DNMT inhibitors have been widely studied in clinical practices and demonstrated synergistic effects in both hematological and solid tumors [55]. Azacytidine (AZA), which is a representative drug in DNMT inhibitors, has been shown to elevate the expression of tumor antigens and immune co-stimulatory molecules, leading to enhanced antigen presentation and increased cytotoxicity of effector T cells [56]. Moreover, this inhibitor can induce an innate immune response via upregulating IFN-α and IFN-β, subsequently increasing the immunogenicity of tumor cells and the efficacy of ICB therapies [57]. Thus, we can conclude that the combination therapy based on AZA and ICB will help to remodel tumor microenvironment in TNBC and thus boost immunotherapy outcomes.

More importantly, in order to reduce the side effects associated with anti-PD-L1 antibody, we propose utilizing CRISPRi system to attenuate PD-L1 expression at transcriptional level. It has several advantages compared to anti-PD-L1 antibody. Firstly, CRISPRi-based modifications can enable more sustained PD-L1 regulation in tumor cells, potentially reducing the need for repeated treatments. Secondly, the genome editing tool allows cell type specific targeting, minimizing off-target effects on healthy tissue, thus decreasing risks of autoimmune complications. Thirdly, the CRISPRi system was more feasible to pair with other gene-modifying strategies for synergistic, multi-pathway therapies. Finally, the technology was cost-effectiveness and it may lower long-term costs. Therefore, the CRISPRi system holds great promise as a precise epigenetic regulatory tool for downregulating PD-L1 expression in TNBC.

In this study, a ROS-responsive polyplex system was designed to co-deliver PD-L1 specific targeting CRISPR/dCas9-KRAB plasmid and the epigenetic inhibitor AZA. AZA was conjugated with PBAE based on the following benefits. Firstly, the ROS-sensitive linkage allows for controlled release of AZA in response to the elevated ROS levels in the tumor cells. This not only reduces the ROS increase caused by the cationic polymer but also enhances gene transfection efficiency. Secondly, compared to free AZA, the conjugated form significantly improves retention at the tumor site, thereby increasing drug utilization. Thirdly, the covalent conjugation via side chains also facilitates the quantification of AZA on the side chains, enabling better control over drug dosing. Among these, the thioether bond in PBAE-S-AZA is the key ROS-responsive trigger. When intracellular ROS levels rise, the thioether bond is oxidized to sulfoxide group, which reduces the polymer's stability and speeds up its degradation [36]. This accelerated breakdown promotes the release of the nucleic acids, making them available for transcription. By scavenging ROS and undergoing thioether-enhanced disassembly, the polymer reduces oxidative stress to protect nucleic acids and promotes their release, thereby enhancing transfection efficiency [38]. Furthermore, other studies have also found that redox-responsive polymers exhibit higher transfection efficiency, which is probably due to the presence of more protonated amines at physiological pH to facilitate DNA condensation [58,59].

This system leveraged the ROS-responsive PBAE-S-AZA cationic polymer prodrug to efficiently encapsulate and deliver the gene cargo to tumor cells. The polyplexes could penetrate into tumor tissues and be taken up by tumor cells efficiently. The thioether bonds in the polyplexes were oxidized and hydrolyzed under high intracellular ROS conditions, thus releasing the plasmid and AZA. The released plasmid was further transcribed and translated into dCas9-KRAB protein and sgRNA, which could specifically target and downregulate PD-L1 expression to relieve immune checkpoint blockade. In the meantime, AZA enhanced antitumor efficacy by increasing MHC class I expression and enhancing antigen presentation. The ROS-responsive polyplex system effectively combined the advantages of gene editing and epigenetic therapy, achieving synergistic effects that convert “cold” tumors into “hot” tumors. This innovative approach not only downregulated PD-L1 expression but also enhanced the overall immunogenicity of the tumor microenvironment.

Moreover, regarding localized administration, this approach remains clinically viable for superficial tumors through methods such as direct injection or the use of an implanted port [60]. Particularly for breast cancer, which is often superficial and accessible, localized delivery can achieve therapeutic drug concentrations while improving the therapeutic window [61]. Additionally, many polymeric systems have been fabricated into a variety of complex structures that are biocompatible and biodegradable, offering unique mechanisms to release therapies at the site of tumors [62]. These make localized administration especially advantageous for breast cancer treatment. Despite its potential, the application of this system for treating deep-seated tumors is still technically challenging due to the polyplexes' positively charged surface. To overcome this, surface modifications, such as carboxylated-mannan coating [63] or biomimetic cell membrane encapsulation [64], could enhance biocompatibility and targeted delivery. Additionally, recent advancements in conjugating hyaluronic acid with trimethylamine oxide have enabled oral delivery of the PBAE-CRISPR–Cas9 system for gastrointestinal tumors [65], offering guidance for advancing PBAE-based systems toward clinical application.

Despite these promising results, several limitations still remain to be solved. Firstly, the PAC polyplex displayed variable toxicity across cell lines; thus, further optimization of dosage and evaluation of potential long-term effects need to be explored. Secondly, how to improve the immunotherapy efficacy in PD-L1-negative tumors requires further investigation. Thirdly, although our cell line–derived xenograft models provided initial insights, they may not fully recapitulate the human immune microenvironment or hematopoietic conditions. Future research will focus on optimizing the delivery system to enhance its efficiency and safety.

## 5 Conclusion

In summary, we developed ROS-responsive polyplexes co-loaded with CRISPRi system and epigenetic inhibitor azacytidine for cancer immunotherapy. The composite polyplexes were demonstrated to have effective cargo packing capacity, high transfection efficiency, superior biosafety, and effective tumor retention. Importantly, the PD-L1 expression in tumor cells could be significantly attenuated by CRISPRi system both *in vitro* and *in vivo*, thus the antitumor immunity was enhanced by releasing the immune checkpoint blockade. Moreover, the AZA co-loaded in the polyplexes could not only improve antigen presentation but also elevate MHC I expression, leading to increased DC maturation and boosted immune response, and thus effectively reshape tumor microenvironment by converting “cold” tumor to “hot” tumor and inhibit tumor growth in TNBC mouse model. Genome editing mediated immune checkpoint blockade combined with epigenetic modulation was proved to be a superior therapeutic method for cancer immunotherapy and provided a potential system for antitumor treatment.

## CRediT authorship contribution statement

**Huan Deng:** Writing – review & editing, Writing – original draft, Formal analysis, Conceptualization. **Qianru Li:** Writing – original draft, Visualization, Validation, Methodology, Formal analysis, Data curation. **Bingxu Wang:** Validation, Methodology. **Hong Yu:** Validation, Methodology. **Shouzheng Sun:** Validation, Methodology. **Zichen Li:** Validation, Methodology. **Weizhen Pan:** Validation, Methodology. **Qianfu Zhao:** Validation, Methodology. **Heshuang Dai:** Validation, Methodology. **Jiao Lu:** Validation, Methodology. **Lihong Fan:** Writing – review & editing, Software, Resources, Data curation, Conceptualization. **Songwei Tan:** Writing – review & editing, Resources, Project administration, Investigation, Conceptualization.

## Funding

This work was supported by the 10.13039/501100001809National Natural Science Foundation of China (82102888, 8210103437, 81871473); the 2025 Provincial Key R&D 10.13039/100020760Sanya Yazhou Bay Science and Technology City Joint Innovation Project (No. ZDYF2025GXJS148); the 2024 Key R&D projects in Hainan Province (No. ZDYF2024GXJS016); the Science and Technology Project of Wuhan (2022023702025187); 10.13039/501100007129Natural Science Foundation of Shandong Province (ZR202306010004); Research projects of the University Institute in the construction period of Shangyu District, Shaoxing City (No: BZLX2023004); the 2022 Sanya Science and Technology Innovation Project (No. 2022KJCX84) and thanks for the supporting by the 2024 Chu Tian Talent Plan Science and Technology Innovation Team Project.

## Declaration of competing interest

The authors declare that they have no known competing financial interests or personal relationships that could have appeared to influence the work reported in this paper.

## Data Availability

Data will be made available on request.

## References

[1] Topalian S.L., Forde P.M., Emens L.A., Yarchoan M., Smith K.N., Pardoll D.M. (2023). Neoadjuvant immune checkpoint blockade: a window of opportunity to advance cancer immunotherapy. Cancer Cell.

[2] Zhu S., Zhang T., Zheng L., Liu H., Song W., Liu D., Li Z., xian Pan C. (2021). Combination strategies to maximize the benefits of cancer immunotherapy. J. Hematol. Oncol..

[3] Choi Y., Seok S.H., Yoon H.Y., Ryu J.H., Kwon I.C. (2024). Advancing cancer immunotherapy through siRNA-based gene silencing for immune checkpoint blockade. Adv. Drug Deliv. Rev..

[4] Wang Y., Gao D., Jin L., Ren X., Ouyang Y., Zhou Y., He X., Jia L., Tian Z., Wu D., Yang Z. (2023). NADPH selective Depletion Nanomedicine-mediated Radio-Immunometabolism regulation for Strengthening anti-PDL1 therapy against TNBC. Adv. Sci. (Weinh.).

[5] Martins F., Sofiya L., Sykiotis G.P., Lamine F., Maillard M., Fraga M., Shabafrouz K., Ribi C., Cairoli A., Guex-Crosier Y., Kuntzer T., Michielin O., Peters S., Coukos G., Spertini F., Thompson J.A., Obeid M. (2019). Adverse effects of immune-checkpoint inhibitors: epidemiology, management and surveillance. Nat. Rev. Clin. Oncol..

[6] Morad G., Helmink B.A., Sharma P., Wargo J.A. (2021). Hallmarks of response, resistance, and toxicity to immune checkpoint blockade. Cell.

[7] Chiappinelli K.B., Strissel P.L., Desrichard A., Li H., Henke C., Akman B., Hein A., Rote N.S., Cope L.M., Snyder A., Makarov V., Buhu S., Slamon D.J., Wolchok J.D., Pardoll D.M., Beckmann M.W., Zahnow C.A., Mergoub T., Chan T.A., Baylin S.B., Strick R. (2015). Inhibiting DNA methylation causes an interferon response in cancer via dsRNA including Endogenous Retroviruses. Cell.

[8] Dear A.E. (2016). Epigenetic Modulators and the new Immunotherapies. N. Engl. J. Med..

[9] Hogg S.J., Beavis P.A., Dawson M.A., Johnstone R.W. (2020). Targeting the epigenetic regulation of antitumour immunity. Nat. Rev. Drug Discov..

[10] Fagbemi O.A., Ojo-omoniyi D.S., Bassey S.I., Ogbozor F.I., Nnamdi O.I., Okei N.C., Kaura S. (2024). Epigenetic therapies in cancer treatment: Opportunities and challenges. World Journal of Biology Phamacy and Health Sciences.

[11] Yang J., Xu J., Wang W., Zhang B., Yu X., Shi S. (2023). Epigenetic regulation in the tumor microenvironment: molecular mechanisms and therapeutic targets. Signal Transduct. Targeted Ther..

[12] Chatterjee A., Rodger E.J., Ahn A., Stockwell P.A., Parry M., Motwani J., Gallagher S.J., Shklovskaya E., Tiffen J., Eccles M.R., Hersey P. (2018). Marked Global DNA Hypomethylation is associated with Constitutive PD-L1 expression in Melanoma. IScience.

[13] Dai E., Zhu Z., Wahed S., Qu Z., Storkus W.J., Guo Z.S. (2021). Epigenetic modulation of antitumor immunity for improved cancer immunotherapy. Mol. Cancer.

[14] Luke J.J., Fakih M., Schneider C., Chiorean E.G., Bendell J., Kristeleit R., Kurzrock R., Blagden S.P., Brana I., Goff L.W., O'Hayer K., Geschwindt R., Smith M., Zhou F., Naing A. (2023). Phase I/II sequencing study of azacitidine, epacadostat, and pembrolizumab in advanced solid tumors. Br. J. Cancer.

[15] Deng H., Tan S., Gao X., Zou C., Xu C., Tu K., Song Q., Fan F., Huang W., Zhang Z. (2020). Cdk5 knocking out mediated by CRISPR-Cas9 genome editing for PD-L1 attenuation and enhanced antitumor immunity. Acta Pharm. Sin. B.

[16] Zhao L., Luo Y., Huang Q., Cao Z., Yang X. (2020). Photo-enhanced CRISPR/Cas9 system enables robust PD-L1 gene disruption in cancer cells and cancer Stem-like cells for efficient cancer immunotherapy. Small.

[17] Lu Q., Chen R., Du S., Chen C., Pan Y., Luan X., Yang J., Zeng F., He B., Han X., Song Y. (2022). Activation of the cGAS-STING pathway combined with CRISPR-Cas9 gene editing triggering long-term immunotherapy. Biomaterials.

[18] Ganesh S., Kim M.J., Lee J., Feng X., Ule K., Mahan A., Krishnan H.S., Wang Z., Anzahaee M.Y., Singhal G., Korboukh I., Lockridge J.A., Sanftner L., Rijnbrand R., Abrams M., Brown B.D. (2024). RNAi mediated silencing of STAT3/PD-L1 in tumor-associated immune cells induces robust anti-tumor effects in immunotherapy resistant tumors. Mol Ther.

[19] Won J.E., Byeon Y., Wi T.I., Lee C.M., Lee J.H., Kang T.H., Lee J.W., Lee Y., Park Y.M., Han H.D. (2022). Immune checkpoint silencing using RNAi-incorporated nanoparticles enhances antitumor immunity and therapeutic efficacy compared with antibody-based approaches. J. Immunother. Cancer.

[20] Zheng R., Zhang L., Parvin R., Su L., Chi J., Shi K., Ye F., Huang X. (2023). Progress and Perspective of CRISPR-Cas9 technology in Translational medicine. Adv. Sci. (Weinh.).

[21] Kumari A., Kaur A., Aggarwal G. (2023). The emerging potential of siRNA nanotherapeutics in treatment of arthritis. Asian J. Pharm. Sci..

[22] Alerasool N., Segal D., Lee H., Taipale M. (2020). An efficient KRAB domain for CRISPRi applications in human cells. Nat. Methods.

[23] Gilbert L.A., Larson M.H., Morsut L., Liu Z., Brar G.A., Torres S.E., Stern-Ginossar N., Brandman O., Whitehead E.H., Doudna J.A., Lim W.A., Weissman J.S., Qi L.S. (2013). CRISPR-mediated modular RNA-guided regulation of transcription in eukaryotes. Cell.

[24] Dominguez A.A., Lim W.A., Qi L.S. (2016). Beyond editing: repurposing CRISPR-Cas9 for precision genome regulation and interrogation. Nat. Rev. Mol. Cell Biol..

[25] Gilbert L.A., Horlbeck M.A., Adamson B., Villalta J.E., Chen Y., Whitehead E.H., Guimaraes C., Panning B., Ploegh H.L., Bassik M.C., Qi L.S., Kampmann M., Weissman J.S. (2014). Genome-scale CRISPR-mediated control of gene repression and activation. Cell.

[26] Stojic L., Lun A.T.L., Mangei J., Mascalchi P., Quarantotti V., Barr A.R., Bakal C., Marioni J.C., Gergely F., Odom D.T. (2018). Specificity of RNAi, LNA and CRISPRi as loss-of-function methods in transcriptional analysis. Nucleic Acids Res..

[27] Gao X., Dong D., Zhang C., Deng Y., Ding J., Niu S., Tan S., Sun L. (2024). Chitosan-functionalized poly(β-amino ester) hybrid system for gene delivery in Vaginal Mucosal Epithelial cells. Pharmaceutics.

[28] Zou W., Huo B., Tu Y., Zhu Y., Hu Y., Li Q., Yu X., Liu B., Tang W., Tan S., Xiao H. (2025). Metabolic reprogramming by chemo-gene co-delivery nanoparticles for chemo-immunotherapy in head and neck squamous cell carcinoma. Acta Biomater..

[29] Tan S., Yuan C., Zhu Y., Chang S., Li Q., Ding J., Gao X., Tian R., Han Z., Hu Z. (2024). Glutathione hybrid poly (beta-amino ester)-plasmid nanoparticles for enhancing gene delivery and biosafety. J. Adv. Res..

[30] Laster D.J., Akel N.S., Hendrixson J.A., James A., Crawford J.A., Fu Q., Berryhill S.B., Thostenson J.D., Nookaew I., O'Brien C.A., Onal M. (2023). CRISPR interference provides increased cell type-specificity compared to the Cre-loxP system. IScience.

[31] Sheng Y., Wang H., Ou Y., Wu Y., Ding W., Tao M., Lin S., Deng Z., Bai L., Kang Q. (2023). Insertion sequence transposition inactivates CRISPR-Cas immunity. Nat. Commun..

[32] Lim X., Zhang C., Chen X. (2024). Advances and applications of CRISPR/Cas-mediated interference in Escherichia coli. Engineering Microbiology.

[33] Kühn M.W.M., Pemmaraju N., Heidel F.H. (2025). The evolving landscape of epigenetic target molecules and therapies in myeloid cancers: focus on acute myeloid leukemia and myeloproliferative neoplasms. Leukemia.

[34] Shi Y., Yu Q., Tan L., Wang Q., Zhu W.H. (2025). Tumor microenvironment-responsive polymer delivery platforms for cancer therapy. Angewandte Chemie - International Edition.

[35] Wei D., Sun Y., Zhu H., Fu Q. (2023). Stimuli-responsive polymer-based Nanosystems for cancer Theranostics. ACS Nano.

[36] Hu D., Li Y., Li R., Wang M., Zhou K., He C., Wei Q., Qian Z. (2024). Recent advances in reactive oxygen species (ROS)-responsive drug delivery systems for photodynamic therapy of cancer. Acta Pharm. Sin. B.

[37] Li Q., Zhu Y., Ding J., Pan W., Hu Y., Lu J., Gao S., Jiang H., Yin Z., Tan S. (2025). Construction of ROS-responsive poly(β-amino ester)-poly(β-thioether ester) copolymer for enhancing gene delivery and gene therapy. Biomacromolecules.

[38] Srinivas U.S., Tan B.W.Q., Vellayappan B.A., Jeyasekharan A.D. (2019). ROS and the DNA damage response in cancer. Redox Biol..

[39] Hua P., Yang D., Chen R., Qiu P., Chen M. (2022). ROS responsive polyethylenimine-based fluorinated polymers for enhanced transfection efficiency and lower cytotoxicity. Bosn. J. Basic Med. Sci..

[40] Yan A., Chen X., He J., Ge Y., Liu Q., Men D., Xu K., Li D. (2023). Phosphorothioated DNA engineered Liposomes as a general platform for Stimuli-responsive cell-specific intracellular delivery and genome editing. Angew Chem. Int. Ed. Engl..

[41] Zhang Z., Qiu N., Wu S., Liu X., Zhou Z., Tang J., Liu Y., Zhou R., Shen Y. (2021). Dose-independent transfection of Hydrophobized polyplexes. Adv Mater.

[42] Tucak-Smajić A., Ruseska I., Letofsky-Papst I., Vranić E., Zimmer A. (2023). Development and characterization of cationic Nanostructured lipid carriers as drug delivery systems for miRNA-27a. Pharmaceuticals.

[43] Forrester S.J., Kikuchi D.S., Hernandes M.S., Xu Q., Griendling K.K. (2018). Reactive oxygen species in metabolic and inflammatory signaling. Circ. Res..

[44] Galliani M., Tremolanti C., Signore G. (2019). Nanocarriers for protein delivery to the cytosol: Assessing the endosomal escape of poly(Lactide-co-Glycolide)-poly(Ethylene imine) nanoparticles. Nanomaterials.

[45] Zheng Y., Sun J., Luo Z., Li Y., Huang Y. (2024). Emerging mechanisms of lipid peroxidation in regulated cell death and its physiological implications. Cell Death Dis..

[46] Lee M.S., Kim N.W., Lee K., Kim H., Jeong J.H. (2013). Enhanced transfection by antioxidative polymeric gene carrier that reduces polyplex-mediated cellular oxidative stress. Pharm. Res..

[47] Topper M.J., Vaz M., Chiappinelli K.B., DeStefano Shields C.E., Niknafs N., Yen R.W.C., Wenzel A., Hicks J., Ballew M., Stone M., Tran P.T., Zahnow C.A., Hellmann M.D., Anagnostou V., Strissel P.L., Strick R., Velculescu V.E., Baylin S.B. (2017). Epigenetic therapy Ties MYC Depletion to Reversing immune evasion and treating lung cancer. Cell.

[48] Tu J., Xu H., Ma L., Li C., Qin W., Chen X., Yi M., Sun L., Liu B., Yuan X. (2022). Nintedanib enhances the efficacy of PD-L1 blockade by upregulating MHC-I and PD-L1 expression in tumor cells. Theranostics.

[49] Stone M.L., Chiappinelli K.B., Li H., Murphy L.M., Travers M.E., Topper M.J., Mathios D., Lim M., Shih I.M., Wang T.L., Hung C.F., Bhargava V., Wiehagen K.R., Cowley G.S., Bachman K.E., Strick R., Strissel P.L., Baylin S.B., Zahnow C.A. (2017). Epigenetic therapy activates type I interferon signaling in murine ovarian cancer to reduce immunosuppression and tumor burden. Proc. Natl. Acad. Sci. U. S. A..

[50] Fang H., Guo Z., Chen J., Lin L., Hu Y., Li Y., Tian H., Chen X. (2021). Combination of epigenetic regulation with gene therapy-mediated immune checkpoint blockade induces anti-tumour effects and immune response in vivo. Nat. Commun..

[51] Fransen M.F., Arens R., Melief C.J.M. (2013). Local targets for immune therapy to cancer: tumor draining lymph nodes and tumor microenvironment. Int. J. Cancer.

[52] Kalergis A.H., Boucheron N., Doucey M.A., Palmieri E., Goyarts E.C., Vegh Z., Luescher I.F., Nathenson S.G. (2001). Efficient T cell activation requires an optimal dwell-time of interaction between the TCR and the pMHC complex. Nat. Immunol..

[53] Harris M.A., Savas P., Virassamy B., O'Malley M.M.R., Kay J., Mueller S.N., Mackay L.K., Salgado R., Loi S. (2024). Towards targeting the breast cancer immune microenvironment. Nat. Rev. Cancer.

[54] So J.Y., Ohm J., Lipkowitz S., Yang L. (2022). Triple negative breast cancer (TNBC): non-genetic tumor heterogeneity and immune microenvironment: emerging treatment options. Pharmacol. Ther..

[55] Nik Amirah Auni N.M.A., Mohd Redzwan N., Fauzi A.N., Yahya M.M., Wong K.K. (2025). Hypomethylating agents as emerging therapeutics for triple-negative breast cancer. Life Sci..

[56] Wong K.K. (2021). DNMT1: a key drug target in triple-negative breast cancer. Semin. Cancer Biol..

[57] Connolly R.M., Li H., Jankowitz R.C., Zhang Z., Rudek M.A., Jeter S.C., Slater S.A., Powers P., Wolff A.C., Fetting J.H., Brufsky A., Piekarz R., Ahuja N., Laird P.W., Shen H., Weisenberger D.J., Cope L., Herman J.G., Somlo G., Garcia A.A., Jones P.A., Baylin S.B., Davidson N.E., Zahnow C.A., Stearns V. (2016). Combination epigenetic therapy in advanced breast cancer with 5-azacitidine and Entinostat: a phase II National cancer Institute/Stand up to cancer study. Clin. Cancer Res..

[58] Ping Y., Wu D., Kumar J.N., Cheng W., Lay C.L., Liu Y. (2013). Redox-responsive hyperbranched poly(amido amine)s with tertiary amino cores for gene delivery. Biomacromolecules.

[59] Cheng W., Wu D., Liu Y. (2016). Michael addition polymerization of Trifunctional amine and acrylic Monomer: a Versatile platform for Development of biomaterials. Biomacromolecules.

[60] Shaha S., Rodrigues D., Mitragotri S. (2024). Locoregional drug delivery for cancer therapy: preclinical progress and clinical translation. J. Contr. Release.

[61] Luo Y., Li J., Hu Y., Gao F., Pak-Heng Leung G., Geng F., Fu C., Zhang J. (2020). Injectable thermo-responsive nano-hydrogel loading triptolide for the anti-breast cancer enhancement via localized treatment based on “two strikes” effects. Acta Pharm. Sin. B.

[62] Woodring R.N., Gurysh E.G., Bachelder E.M., Ainslie K.M. (2023). Drug delivery systems for localized cancer combination therapy. ACS Appl. Bio Mater..

[63] Chen Y., Chen X., Zhang Y., Wang M., Yang M., Wang R., Yan X., Shao S., Xin H., Hu Q., Wei W., Ping Y. (2025). Macrophage-specific in vivo RNA editing promotes phagocytosis and antitumor immunity in mice. Sci. Transl. Med..

[64] Yan X., Pan Q., Xin H., Chen Y., Ping Y. (2021). Genome-editing prodrug: targeted delivery and conditional stabilization of CRISPR-Cas9 for precision therapy of inflammatory disease. Sci. Adv..

[65] Zhao K., Yan Y., Jin X.K., Pan T., Zhang S.M., Yang C.H., Rao Z.Y., Zhang X.Z. (2025). An orally administered gene editing nanoparticle boosts chemo-immunotherapy in colorectal cancer. Nat. Nanotechnol..

