# Supplementary material for: Reshaping tumor immune microenvironment through ROS-responsive prodrug polyplexes via synergistic effect of CRISPRi system and epigenetic inhibitor for breast cancer therapy
Source: Mater Today Bio. 2025 Sep 4;35:102285. doi: 10.1016/j.mtbio.2025.102285 (PMC12454885; doi:10.1016/j.mtbio.2025.102285)
Supplement: Multimedia component 1 [file mmc1.docx]

Supplementary material

**Reshaping tumor immune microenvironment through**

**ROS-responsive** **prodrug polyplexes via**

**synergistic effect of CRISPRi system and epigenetic inhibitor for breast cancer therapy**

Authors: Huan Deng^1#^, Qianru Li^2,3#^, Bingxu Wang^1^, Hong Yu^1^, Shouzheng Sun^1^, Zichen Li^1^, Weizhen Pan^2^, Qianfu Zhao^1^, Heshuang Dai^1^, Jiao Lu^2^, Lihong Fan^1^*, Songwei Tan^2,3^*

#: These authors contributed equally to this work.

*: Corresponding authors.

Affiliations: ^1^ School of Chemistry, Chemical Engineering and Life Sciences, Wuhan University of Technology, Wuhan, 430070, China.

^2^ School of Pharmacy, Tongji Medical College, Huazhong University of Science and Technology, Wuhan, 430030, China.

^3^ Hubei Provincial Key Laboratory of Pediatric Genetic Metabolic and Endocrine Rare Diseases, Wuhan, 430030, China

Emails：

Songwei Tan, tansw@hust.edu.cn

Lihong Fan, lhfan@whut.edu.cn

Huan Deng, Denghuan@whut.edu.cn

Qianru Li, m202375837@hust.edu.cn

Bingxu Wang, 338843@whut.edu.cn

Hong Yu, 353046@whut.edu.cn

Shouzheng Sun, 367168@whut.edu.cn

Zichen Li, 1762815537@qq.com

Weizhen Pan, panweizhen@hust.edu.cn

Qianfu Zhao, zhaoqianfu2024@whut.edu.cn

Heshuang Dai, daiheshuang@whut.edu.cn

Jiao Lu, 3531384808@qq.com

**This file contains：**

**Figure S1.** Design and synthesis of PBAE-S-AZA.

**Figure S2.** ^1^H NMR spectrum of PBAE-S-AZA, aPBAE=, aPBAE, AZA-SH, and AZA in DMSO-d_6_.

**Figure S3.** Mass spectra of AZA and AZA-SH (scale bar: m/z).

**Figure S4.** Size distribution of PBAE-S-AZA/pMax-GFP polyplexes at the mass ratio of 50:1.

**Figure S5.** Screening of sgRNA for PD-L1 downregulation in 4T1 cells.

**Figure S6.** Sequencing analysis of the CRISPRi plasmids cloned with designed sgRNAs.

**Figure S7.** Quantitative analysis of PD-L1 expression on 4T1 cells treated with different concentrations of AZA by flow cytometry (n = 3).

**Figure S8.** Flow cytometry migration analysis of the Ce6@PAC polyplexes internalized into 4T1 cells at different time points.

**Figure S9.** Quantification of 4T1 cell viability using the CCK-8 assay.

**Figure S10.** Quantification of B16F10 cell viability using the CCK-8 assay.

**Figure S11.** Transfection efficiency of PBAE-S-AZA/pMax-GFP polyplexes at different mass ratios in B16F10 cells.

**Figure S12.** Transfection efficiency of PBAE-S-AZA/pMax-GFP polyplexes at different mass ratios in N2a cells.

**Figure S13.** Representative flow cytometry gating strategies for activated DCs (CD11c^+^CD80^+^CD86^+^) in BMDCs.

**Figure S14.** Relative quantification of (A) PD-L1 and (B) representative immune related cytokines in 4T1 cells at the transcriptomic level (n = 3, ***p*<0.01, *****p*<0.0001).

**Figure S15.** The immunohistochemical staining of CD31 in tumor tissues from 4T1 tumor-bearing mice after different treatments (scale bar: 100 μm).

**Figure S16.** Body weight changes in different treatment groups during the 4T1 tumor suppression experiment (n = 6).

**Figure S17.** Body weight changes in different treatment groups of the 4T1 tumor-bearing mice during the survival analysis experiment (n = 12).

**Figure S18.** HE staining of major organs (heart, liver, spleen, lung, and kidney) collected from 4T1 tumor-bearing mice after different treatments.

**Figure S19.** The biochemistry parameters (A) alanine aminotransferase (ALT), (B) aspartate transaminase (AST), and (C) blood urea nitrogen (BUN) of blood serum from the 4T1 tumor-bearing mice after different treatments (n = 5).

**Figure S20.** Representative flow cytometry gating strategies for activated DCs (CD11c^+^CD80^+^CD86^+^) in TDLNs of 4T1 tumor-bearing mice.

**Figure S21.** Representative flow cytometry gating strategies for CD8^+^ T cells, CD4^+^ T cells, and Tregs (CD4^+^CD25^+^Foxp3^+^) in tumor tissue of 4T1 tumor-bearing mice.

**Figure S22.** Representative flow cytometry gating strategies for M1 (CD11b^+^F4/80^+^CD86^+^) and M2 (CD11b^+^F4/80^+^CD206^+^) macrophages in tumor tissue of 4T1 tumor-bearing mice.

**Table S1.** Particle size and ζ-potential of PBAE-S-AZA/pMax-GFP polyplexes.

**Table S2.** The sequences of sgRNA oligos used for CRISPR/dCas9-KRAB system.

**
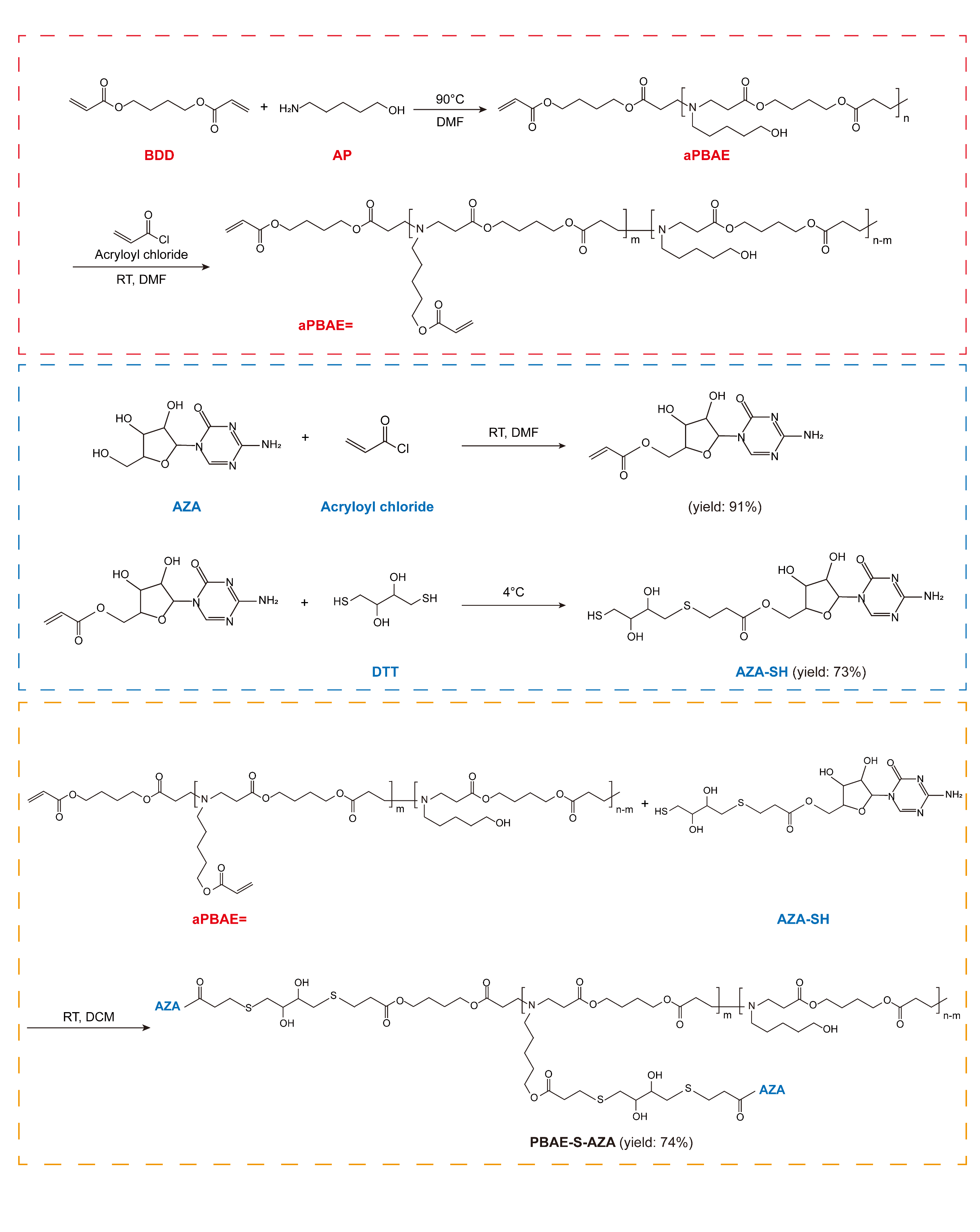
Figure S1.** Design and synthesis of PBAE-S-AZA.

**
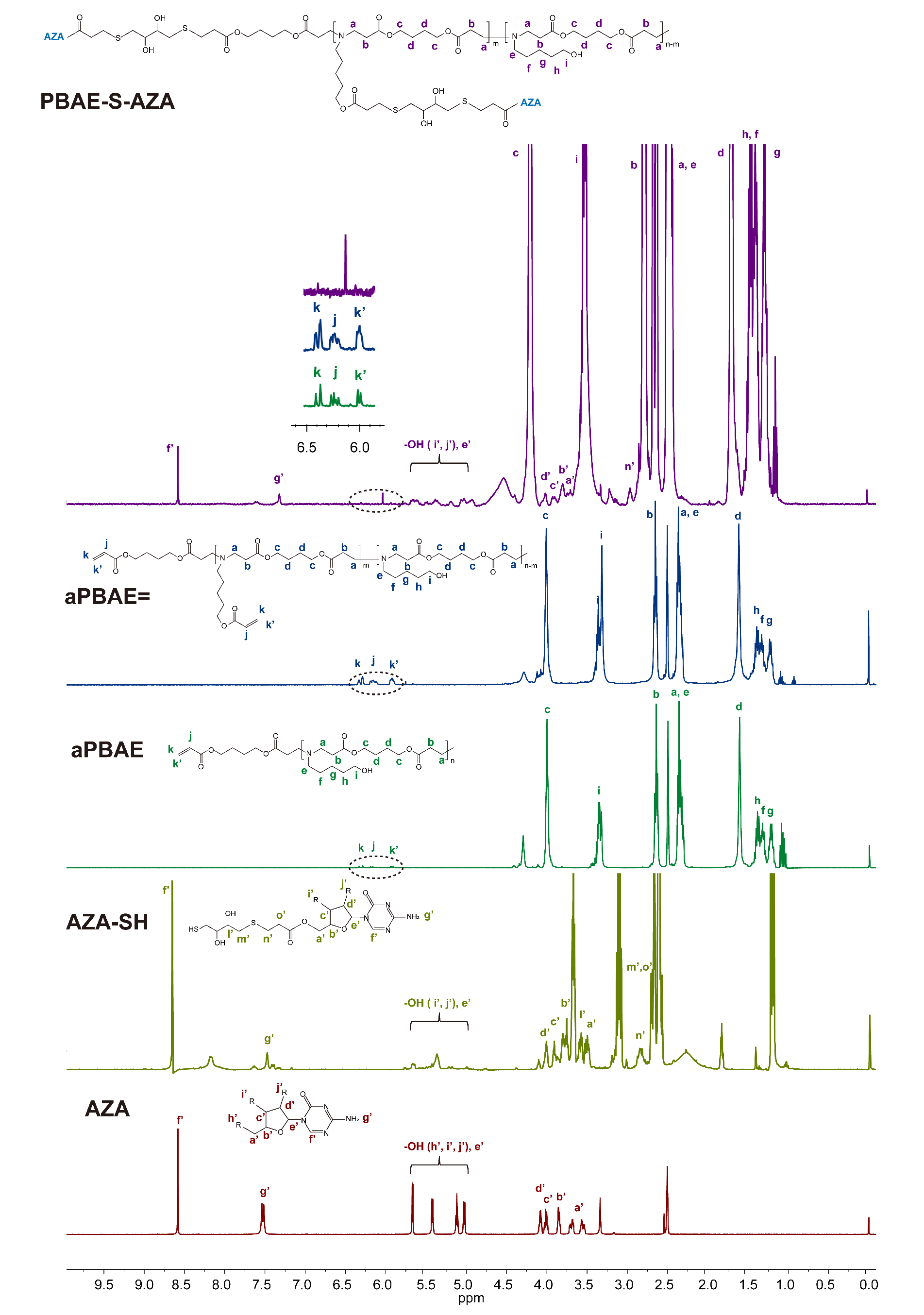
**

**Figure S2.** ^1^H NMR spectrum of PBAE-S-AZA, aPBAE=, aPBAE, AZA-SH, and AZA in DMSO-d_6_.

**
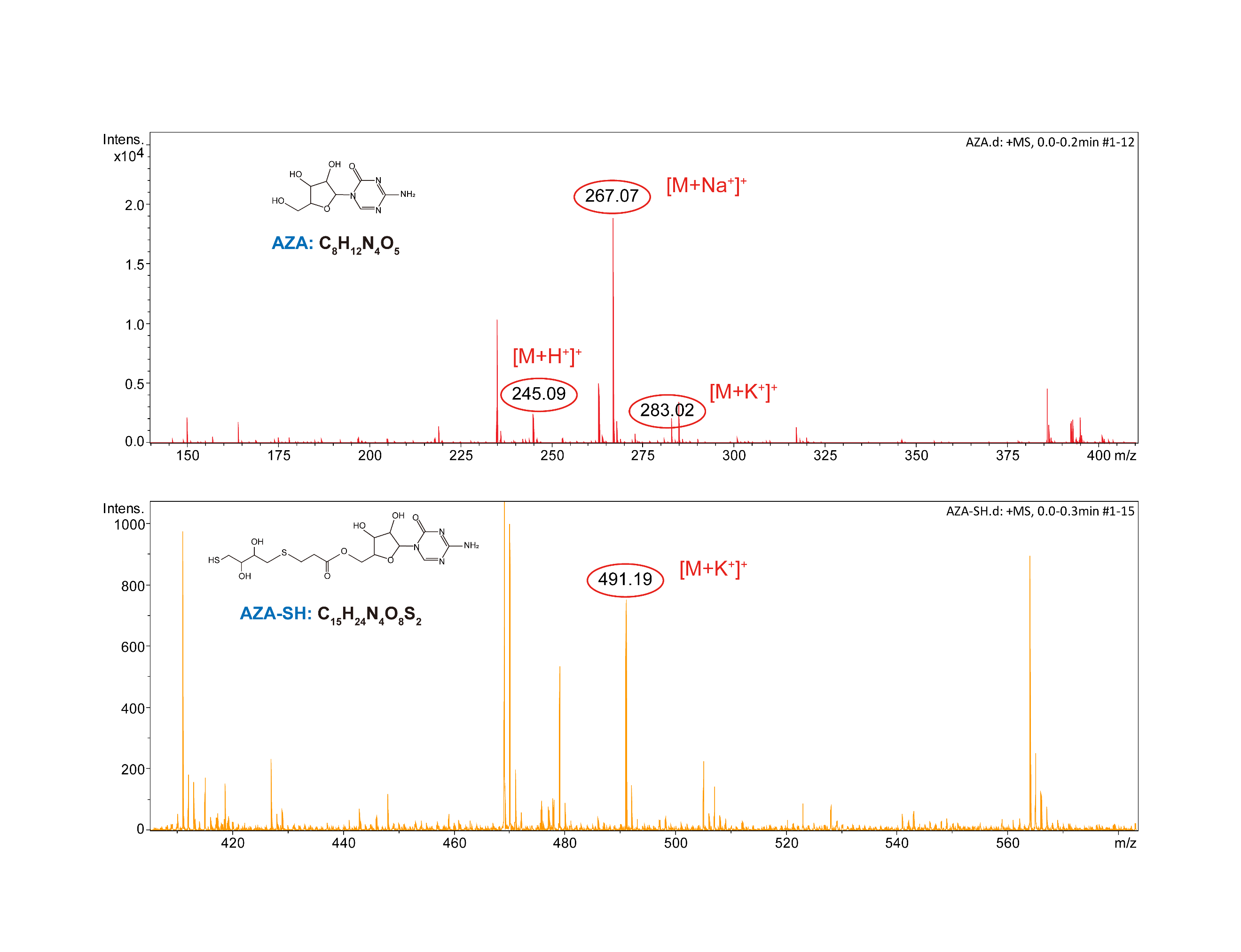
**

**Figure S3.** Mass spectra of AZA and AZA-SH (scale bar: m/z).

**
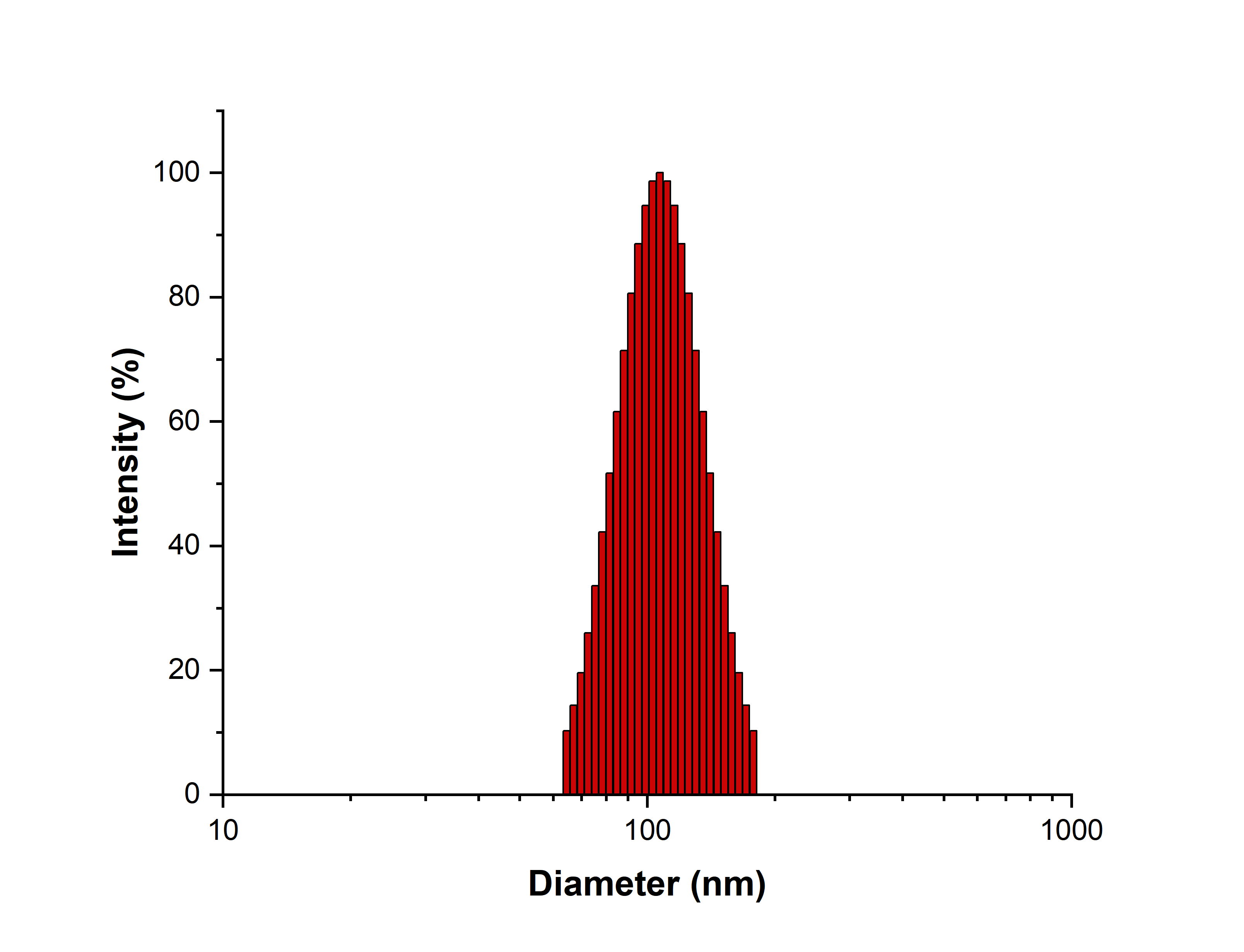
**

**Figure S4.** Size distribution of PBAE-S-AZA/pMax-GFP polyplexes at the mass ratio of 50:1.

**
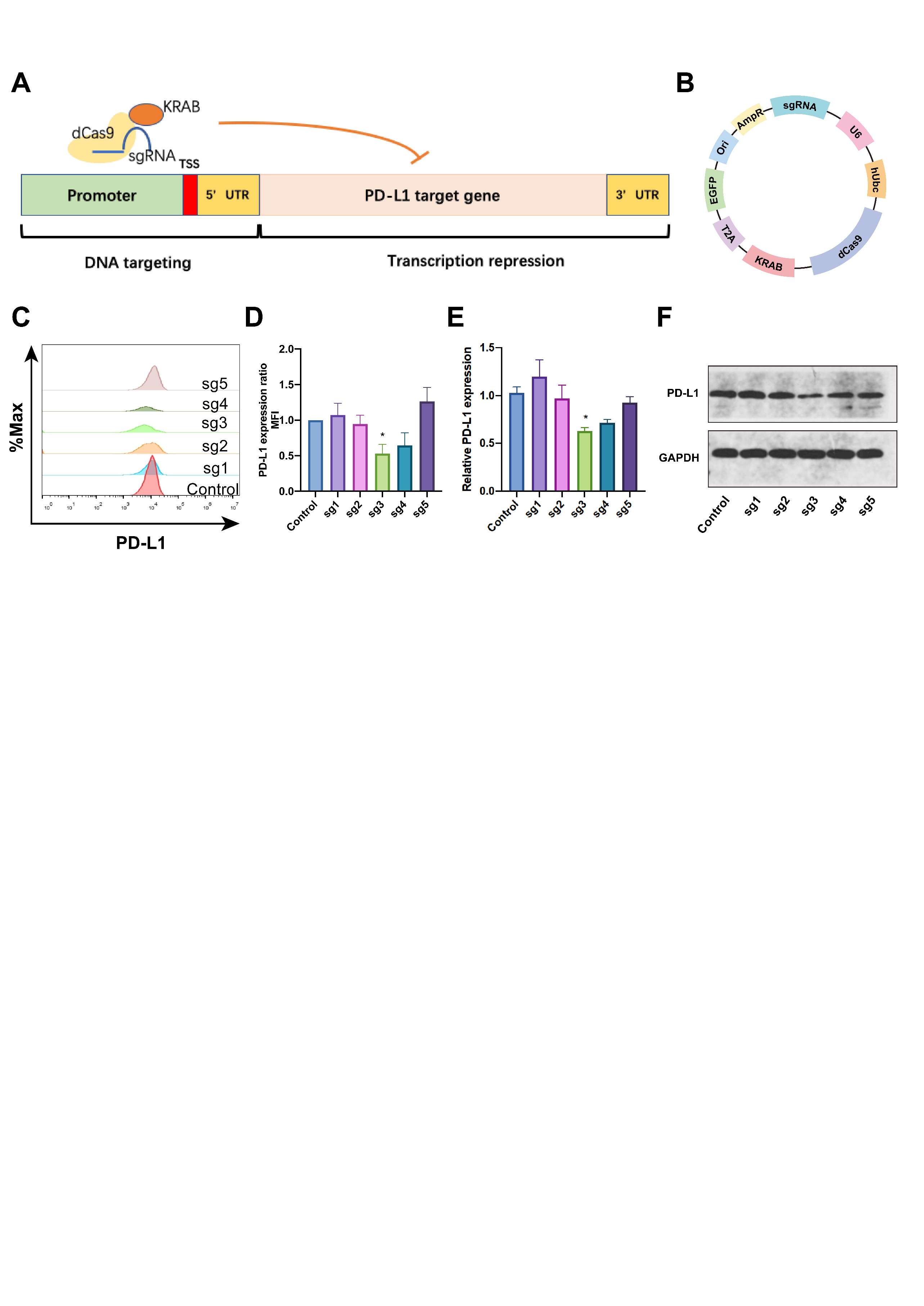
Figure S5.** Screening of sgRNA for PD-L1 downregulation in 4T1 cells. (A) Schematic diagram of dCas9-KRAB/sgRNA targeting PD-L1 gene. (B) Schematic diagram of the CRISPR/dCas9-KRAB plasmid. (C) Flow cytometry migration analysis and (D) quantitative analysis of PD-L1 expression in 4T1 cells transfected with various sgRNAs (n = 3, **p*<0.05). (E) RT-qPCR (n = 3, **p*<0.05) and (F) western blotting of PD-L1 expression in 4T1 cells transfected with various sgRNAs.


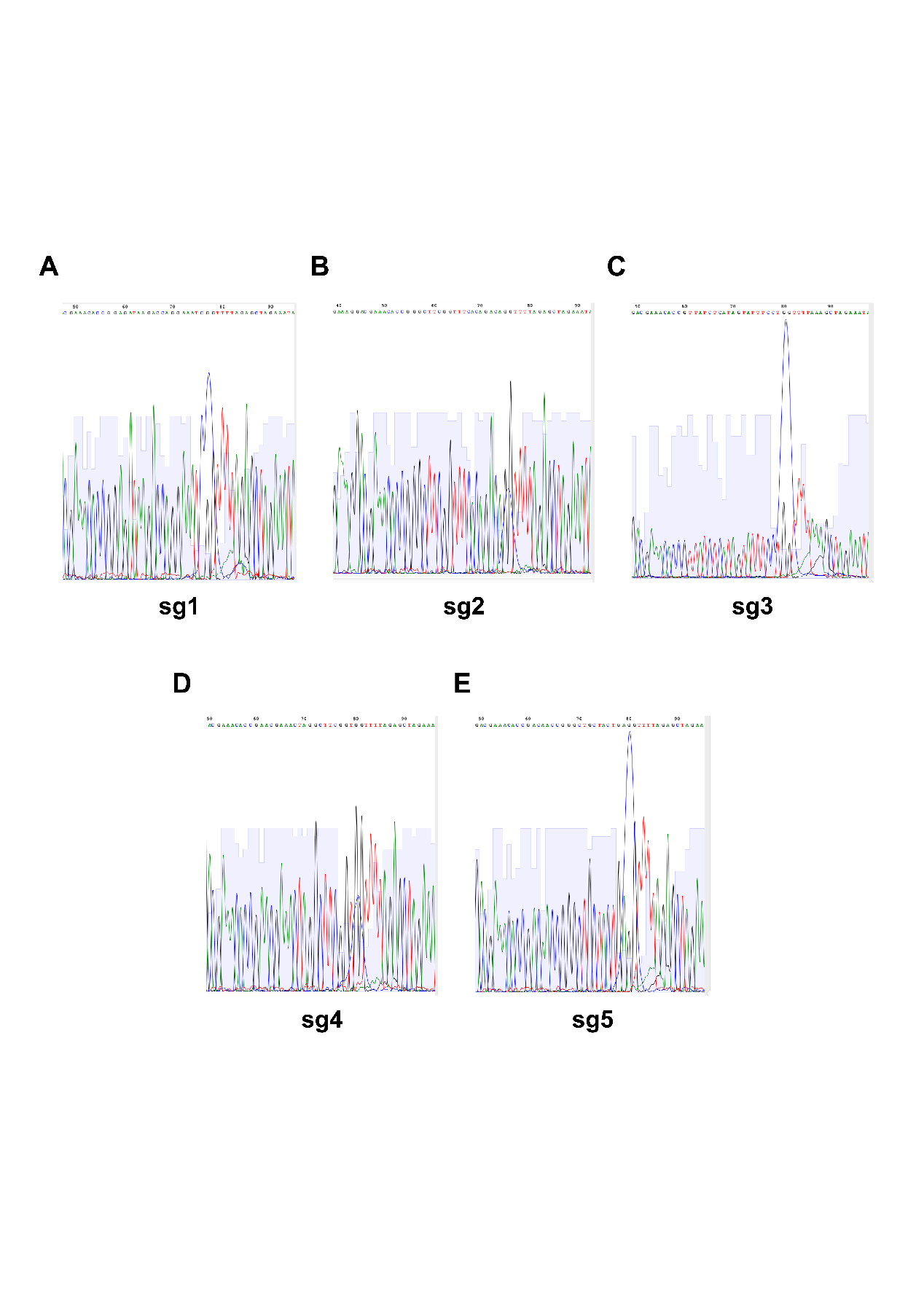


**Figure S6.** Sequencing analysis of the CRISPRi plasmids cloned with designed sgRNAs.


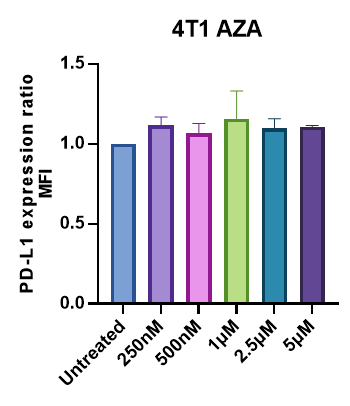


**Figure S7.** Quantitative analysis of PD-L1 expression on 4T1 cells treated with different concentrations of AZA by flow cytometry (n = 3).


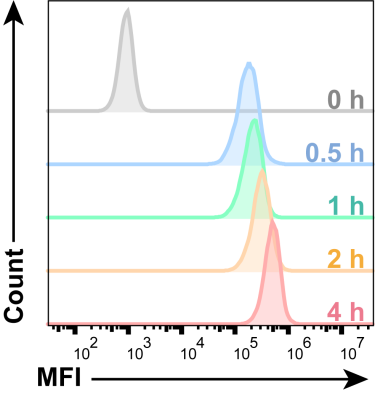


**Figure S8.** Flow cytometry migration analysis of the Ce6@PAC polyplexes internalized into 4T1 cells at different time points.


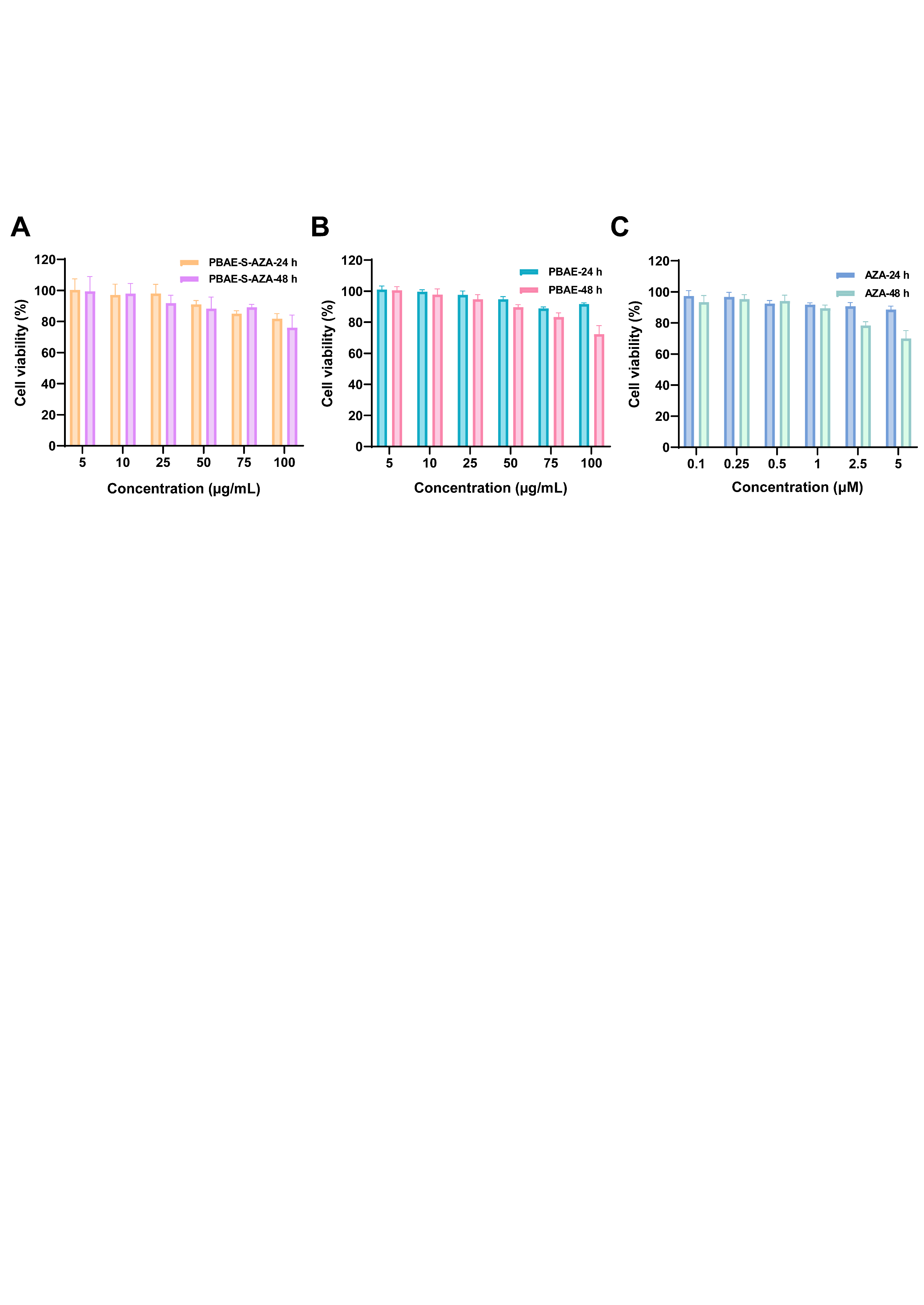


**Figure S9.** Quantification of 4T1 cell viability using the CCK-8 assay. Viability of 4T1 cells after incubation with (A) PBAE-S-AZA polymer, (B) PBAE polymer, and (C) AZA at different concentrations for 24 h or 48 h (n = 5).


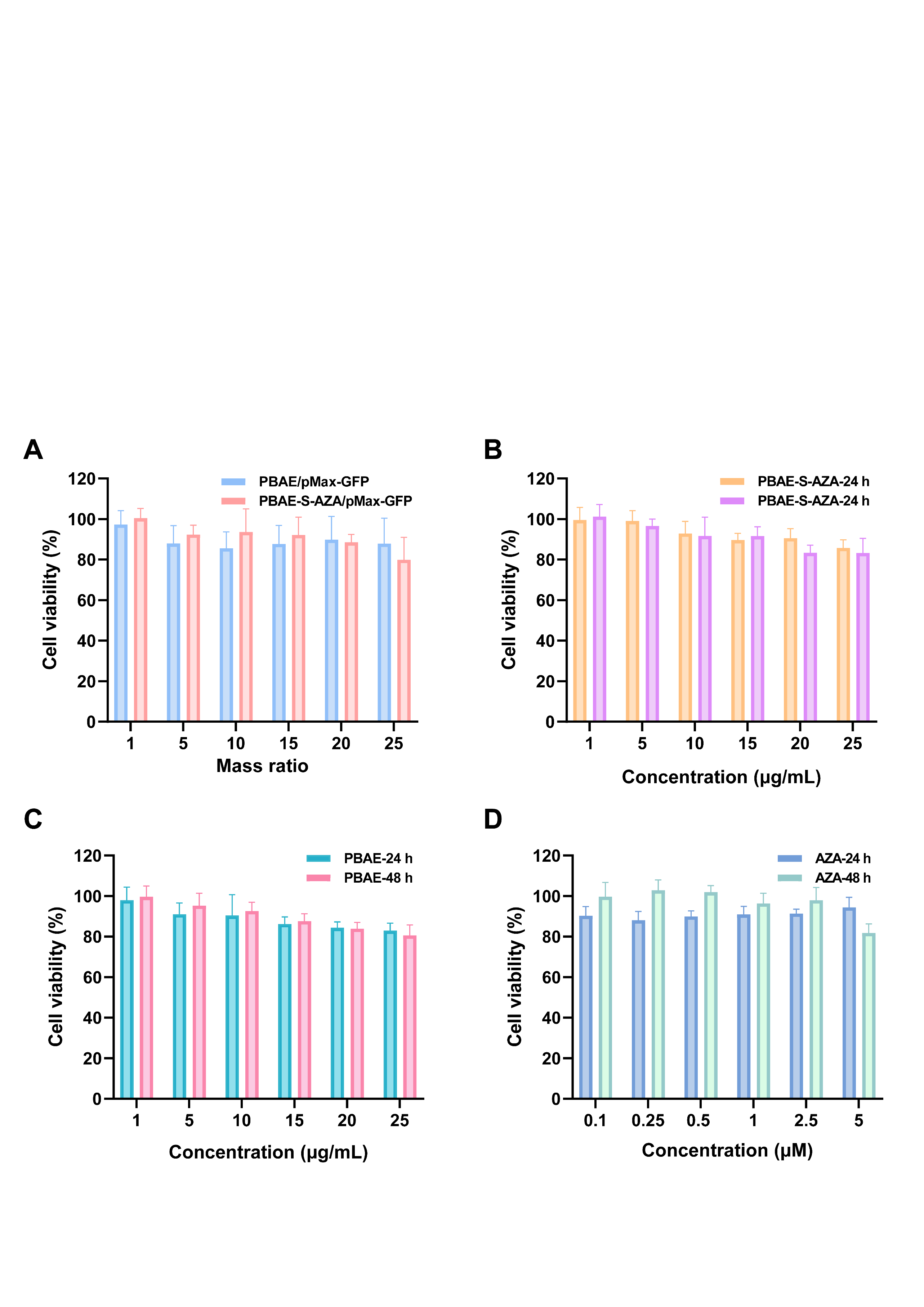


**Figure S10.** Quantification of B16F10 cell viability using the CCK-8 assay. (A) Viability of B16F10 cells after incubation with PBAE/pMax-GFP polyplexes or PBAE-S-AZA/pMax-GFP polyplexes at different mass ratios for 24 h (n = 5). Viability of B16F10 cells after incubation with (B) PBAE-S-AZA polymer, (C) PBAE polymer, and (D) AZA at different concentrations for 24 h or 48 h (n = 5).


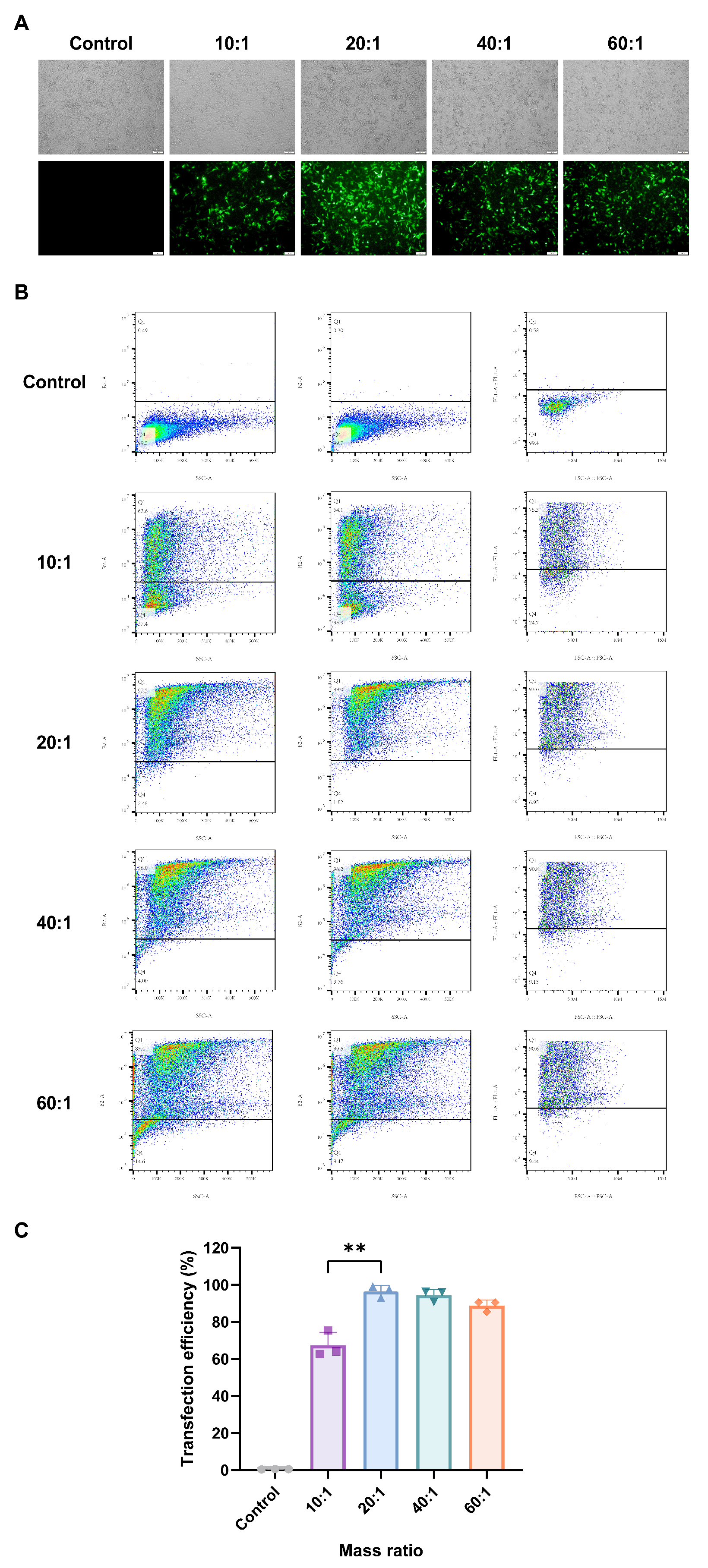


**Figure S11.** Transfection efficiency of PBAE-S-AZA/pMax-GFP polyplexes at different mass ratios in B16F10 cells. (A) Representative fluorescence microscopy images of GFP expression in B16F10 cells transfected with PBAE-S-AZA/pMax-GFP polyplexes at different mass ratios (Scale bar: 100 μm). (B) Flow cytometry scatter plot and (C) quantitative analysis of transfection efficiency in B16F10 cells determined by flow cytometry (n = 3, ***p*<0.01).


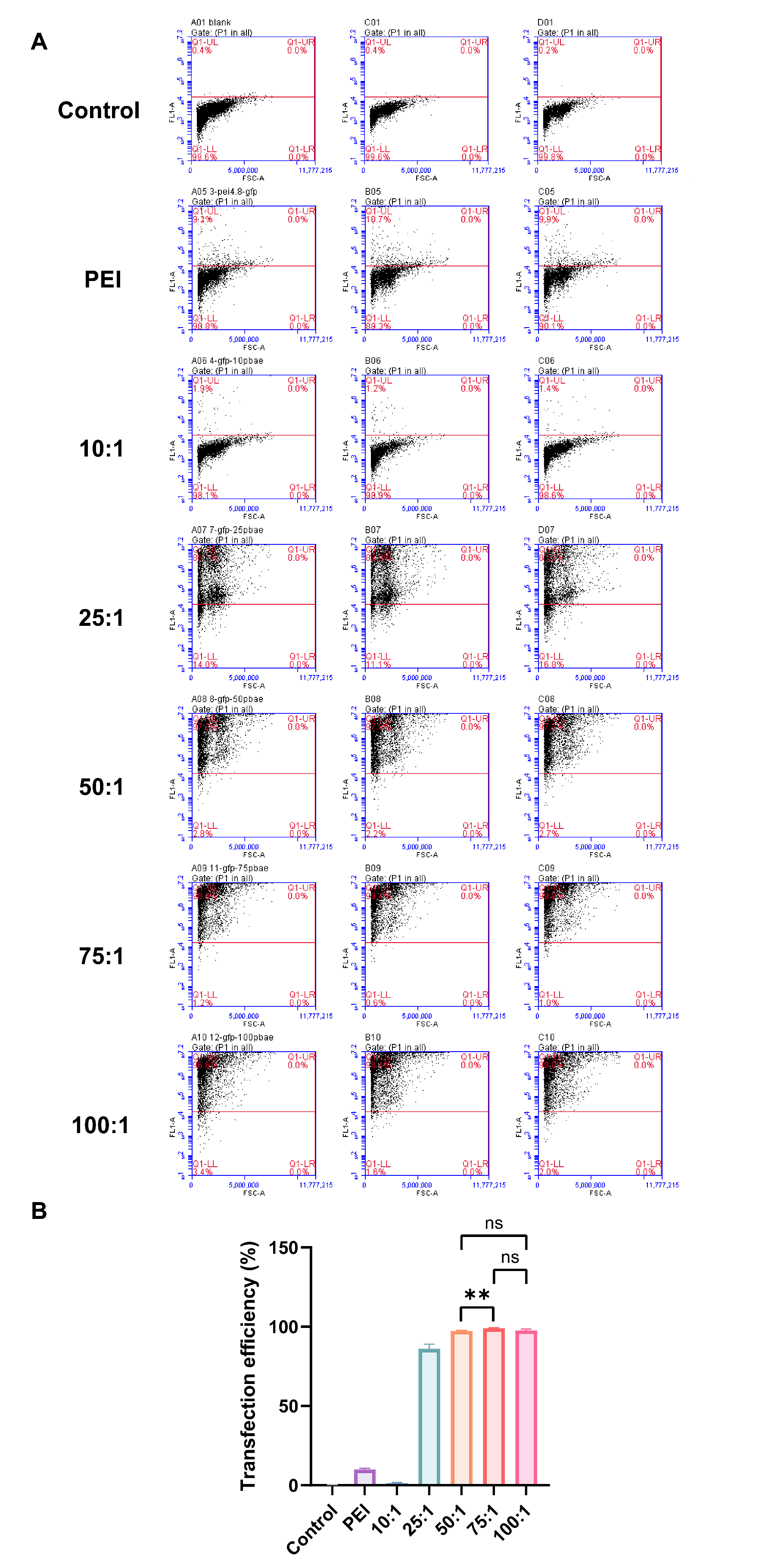


**Figure S12.** Transfection efficiency of PBAE-S-AZA/pMax-GFP polyplexes at different mass ratios in N2a cells. (A) Flow cytometry scatter plot and (B) quantitative analysis of transfection efficiency in N2a cells determined by flow cytometry (n = 3, ***p*<0.01).


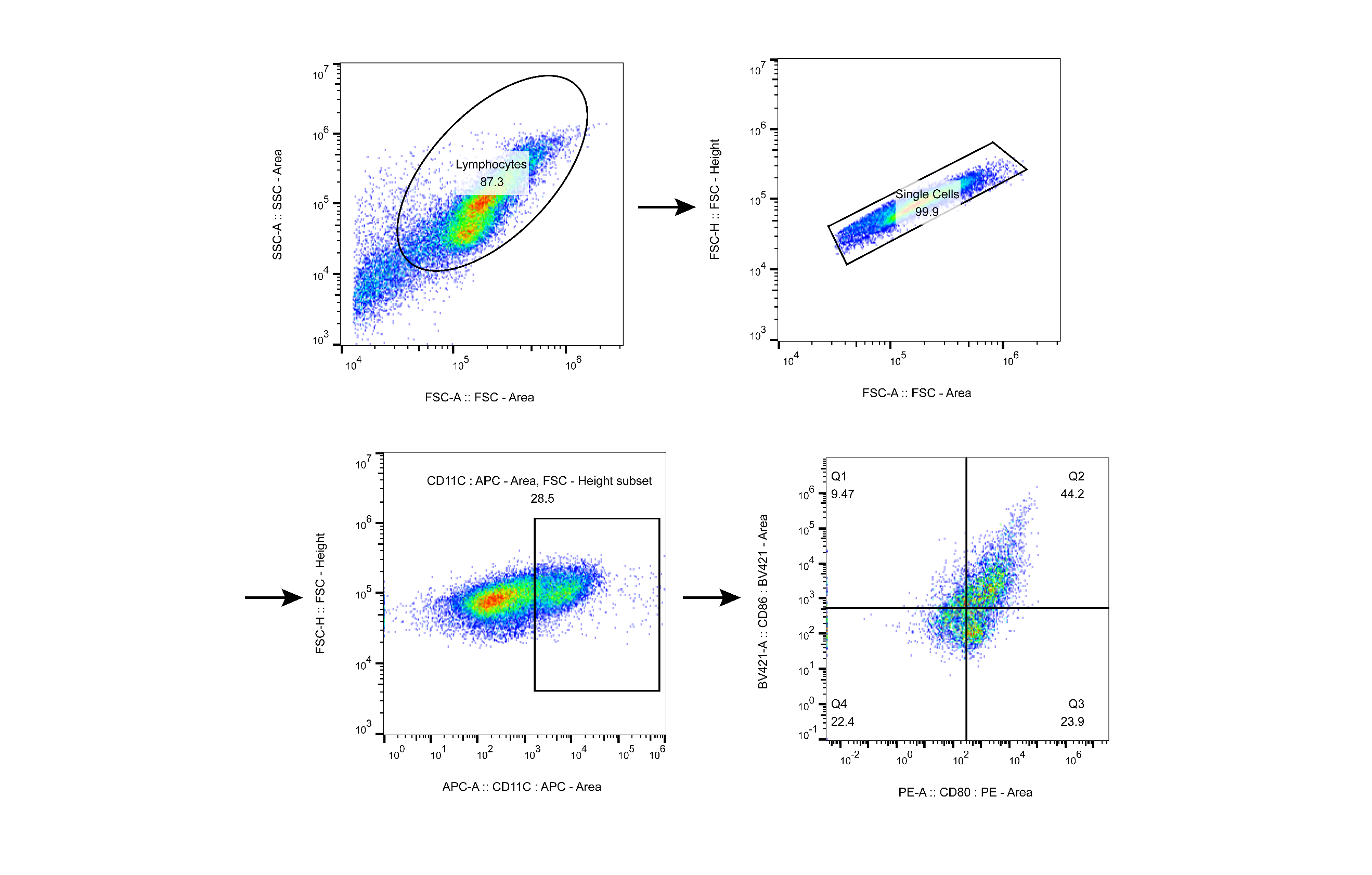


**Figure S13.** Representative flow cytometry gating strategies for activated DCs (CD11c^+^CD80^+^CD86^+^) in BMDCs.


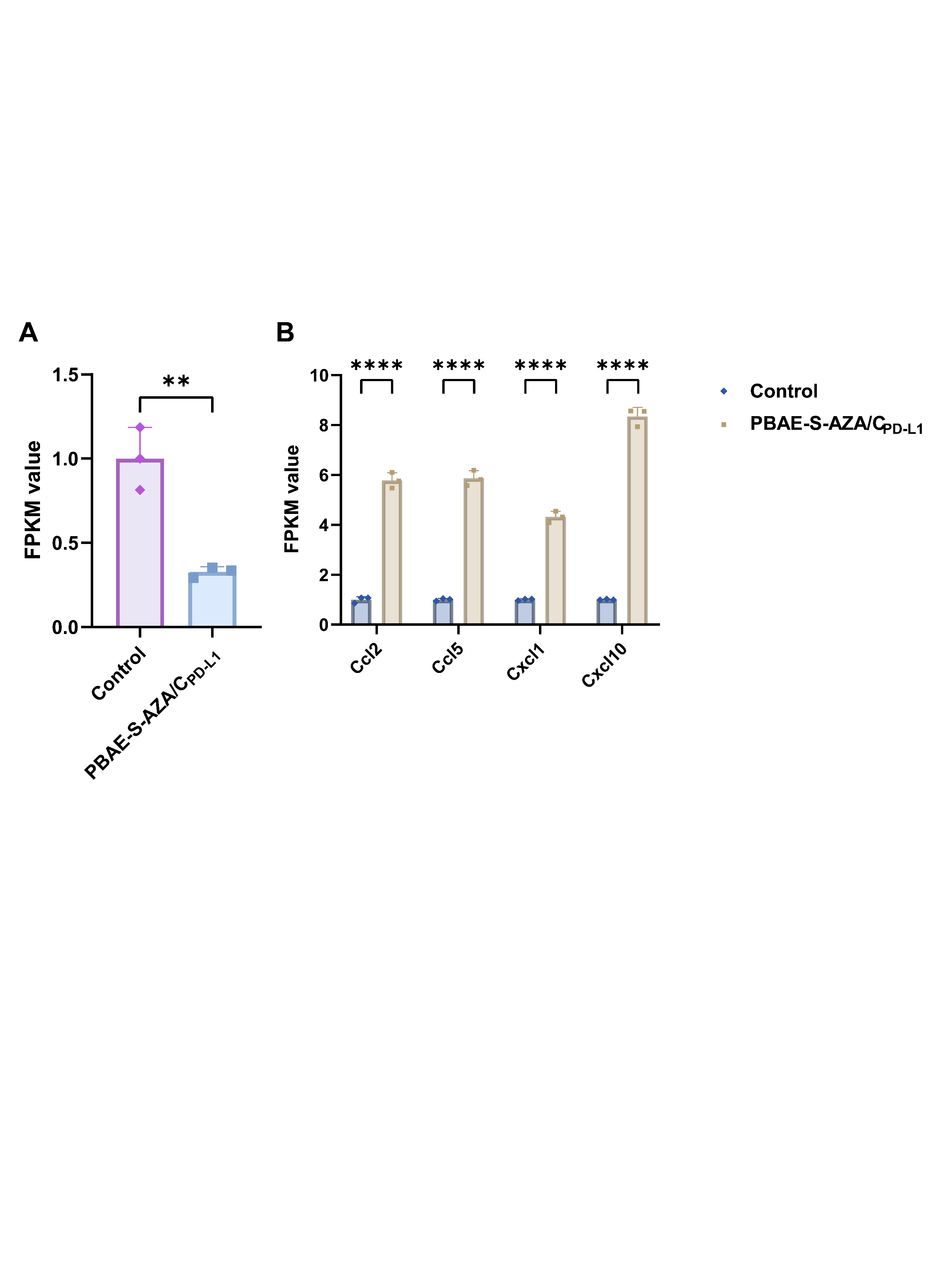


**Figure S14.** Relative quantification of (A) PD-L1 and (B) representative immune related cytokines in 4T1 cells at the transcriptomic level (n = 3, ***p*<0.01, *****p*<0.0001).


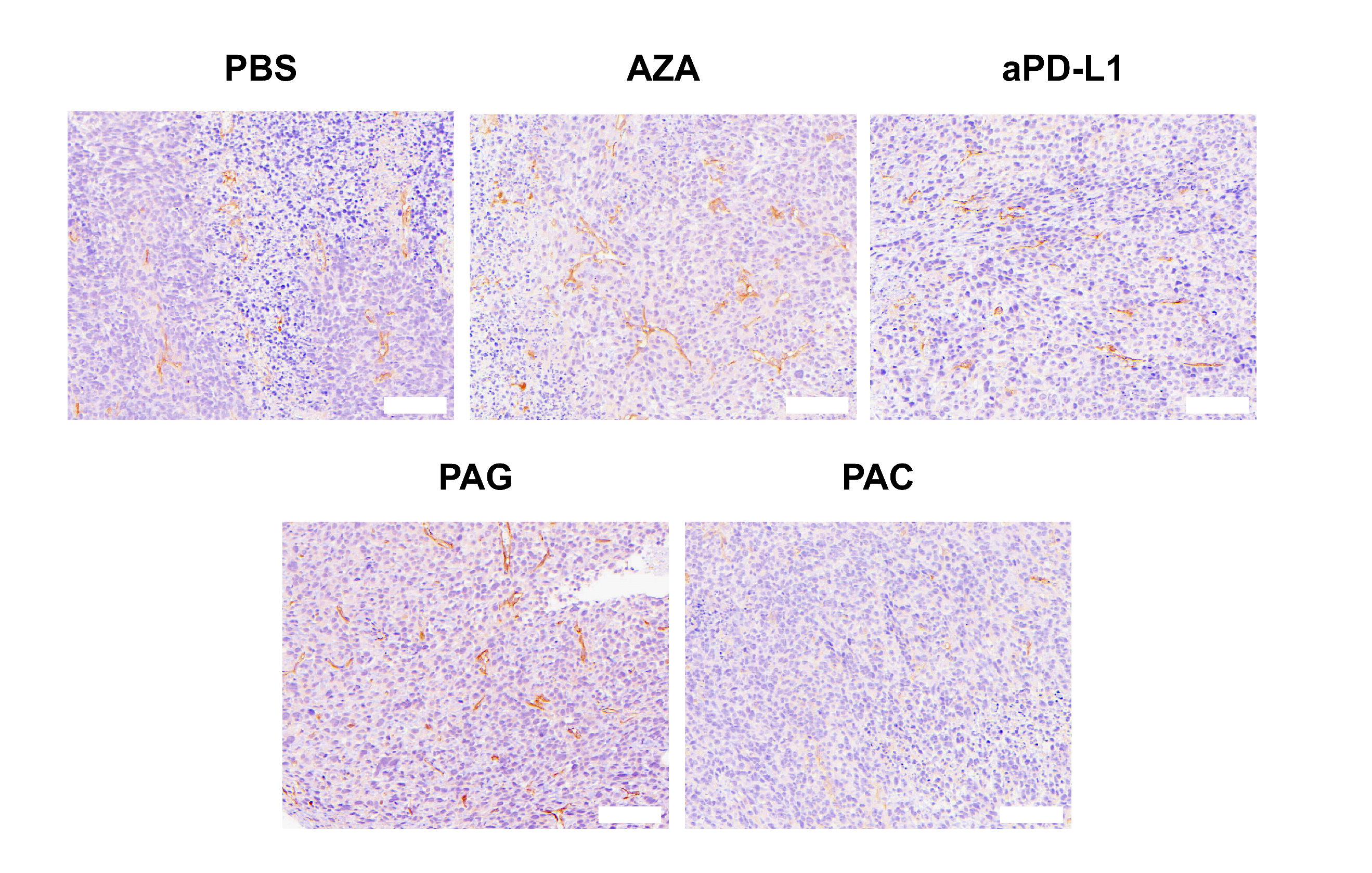


**Figure S15.** The immunohistochemical staining of CD31 in tumor tissues from 4T1 tumor-bearing mice after different treatments (scale bar: 100 μm).

**Figure S16.** Body weight changes in different treatment groups during the 4T1 tumor suppression experiment (n = 6).

**Figure S17.** Body weight changes in different treatment groups of the 4T1 tumor-bearing mice during the survival analysis experiment (n = 12).


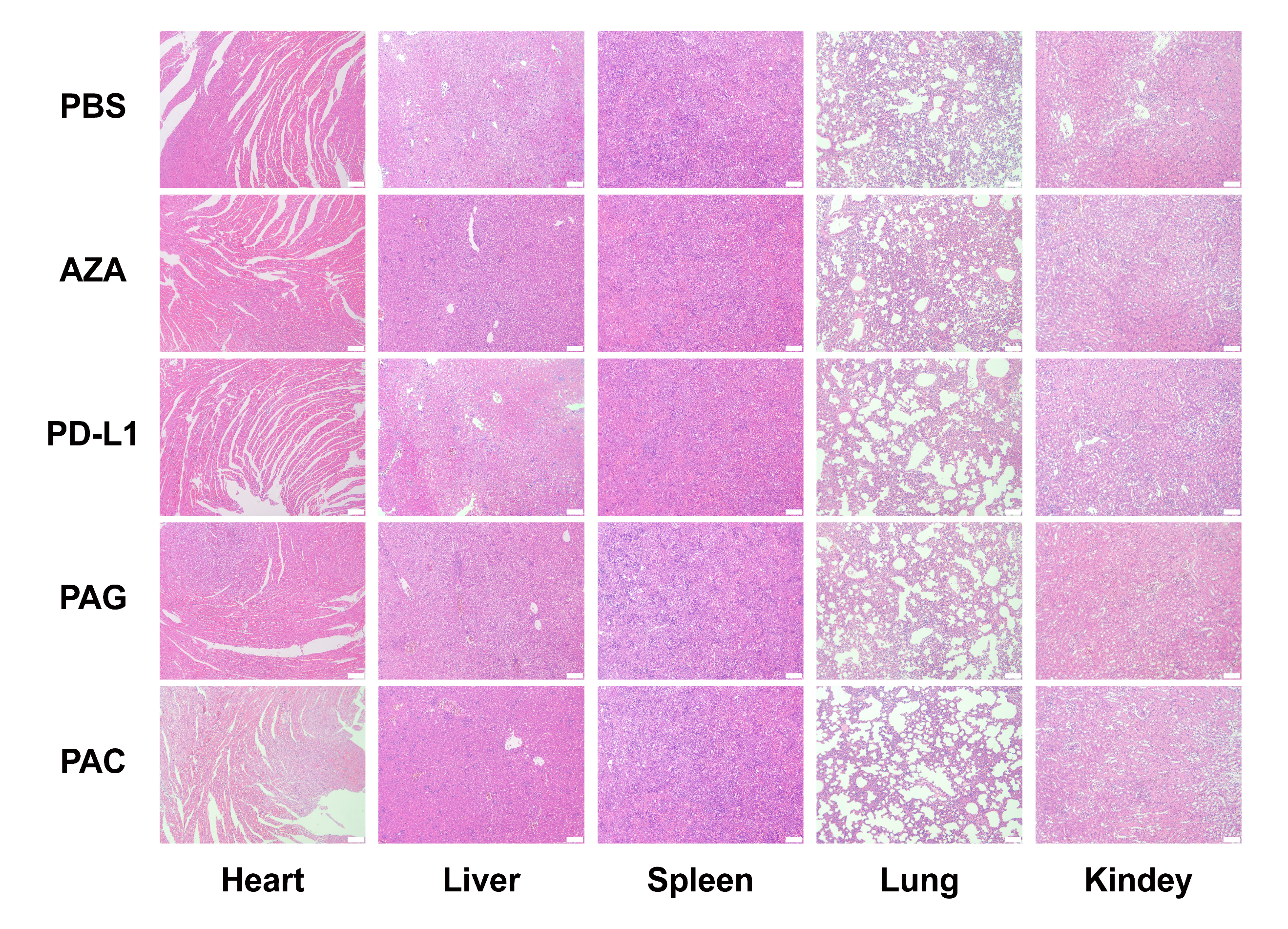


**Figure S18.** HE staining of major organs (heart, liver, spleen, lung, and kidney) collected from 4T1 tumor-bearing mice after different treatments (scale bar: 100 μm).


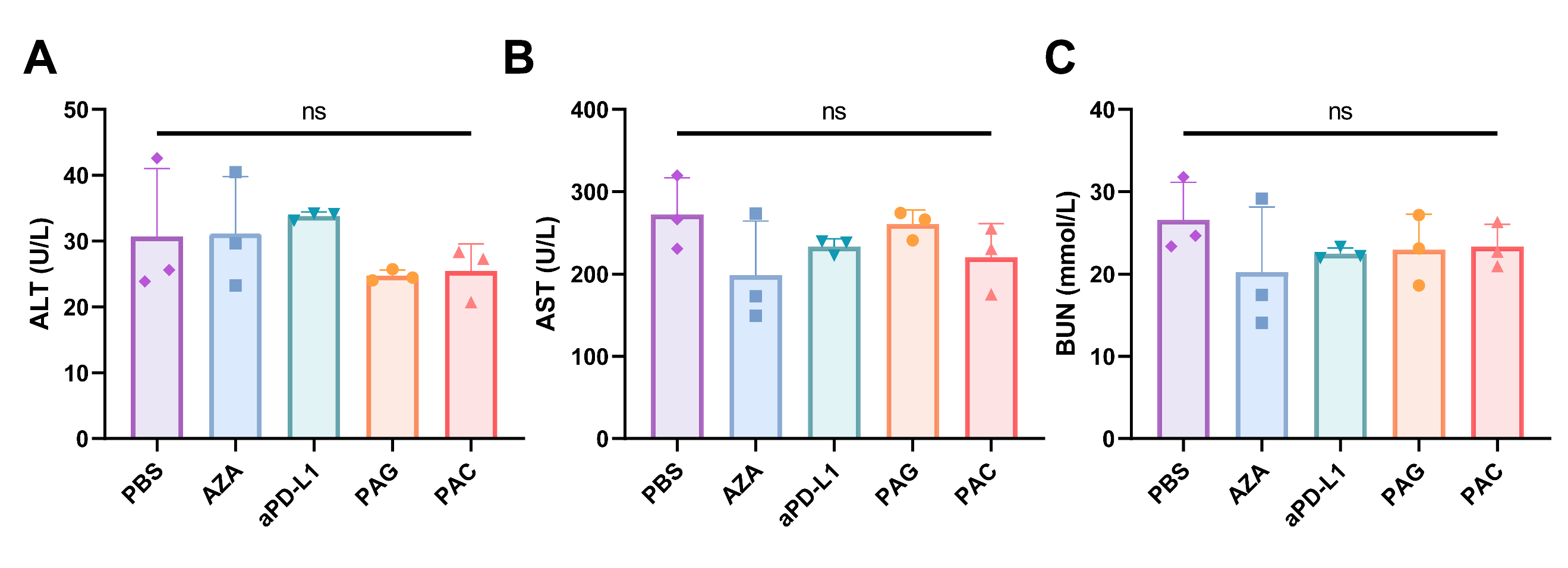


**Figure S19.** The biochemistry parameters (A) alanine aminotransferase (ALT), (B) aspartate transaminase (AST), and (C) blood urea nitrogen (BUN) of blood serum from the 4T1 tumor-bearing mice after different treatments (n = 5).


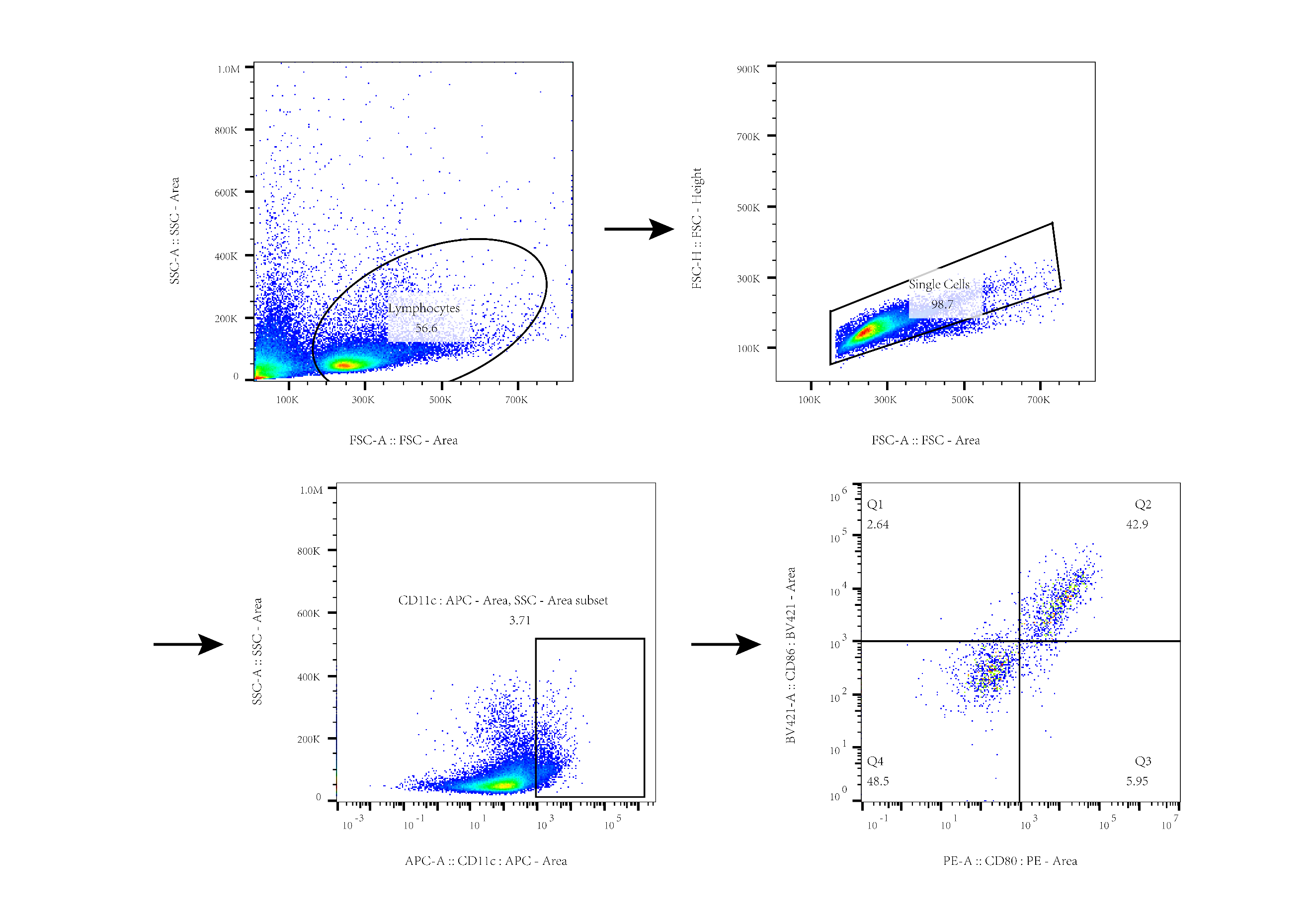


**Figure S20.** Representative flow cytometry gating strategies for activated DCs (CD11c^+^CD80^+^CD86^+^) in TDLNs of 4T1 tumor-bearing mice.


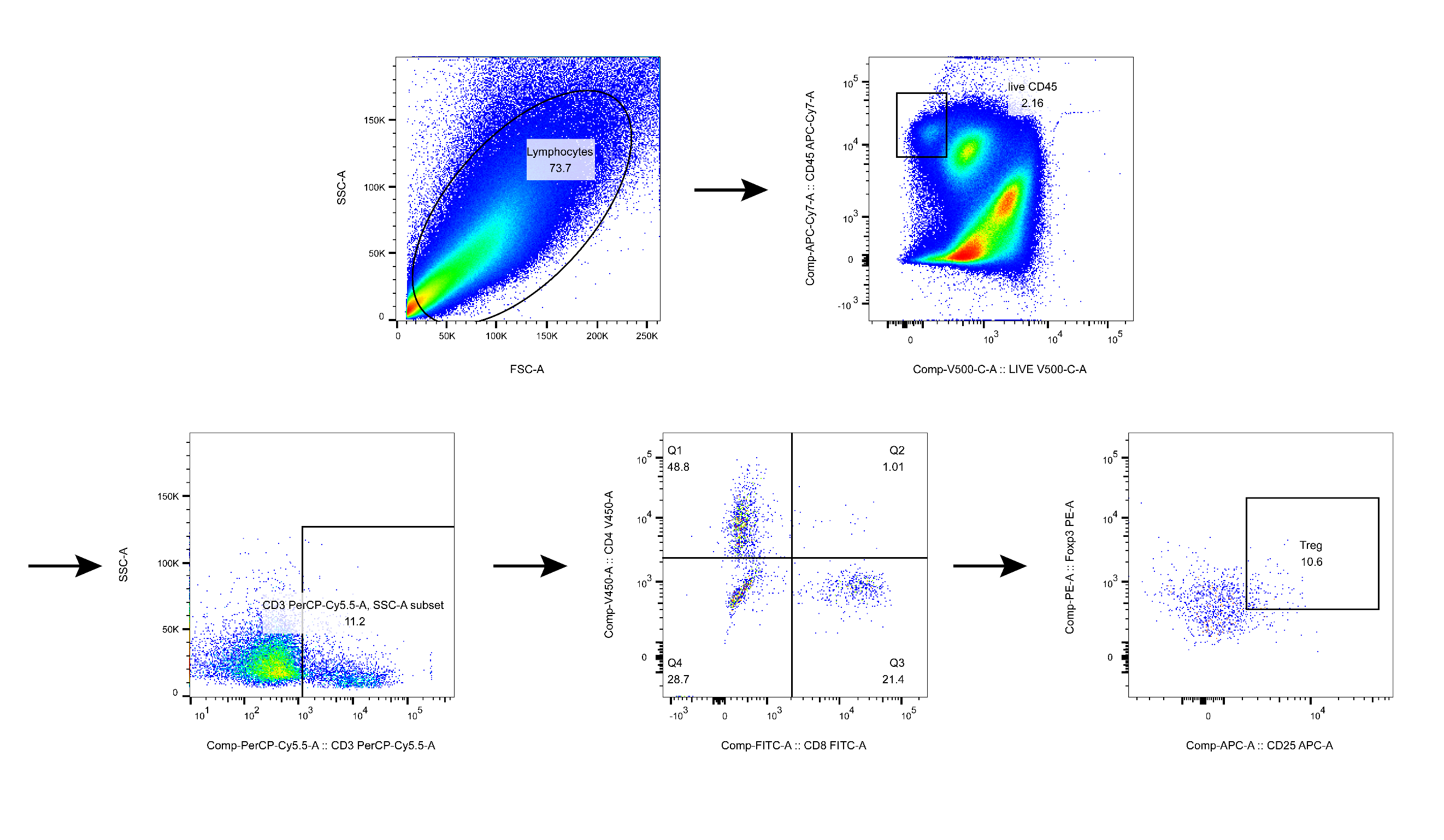


**Figure S21.** Representative flow cytometry gating strategies for CD8^+^ T cells, CD4^+^ T cells, and Tregs (CD4^+^CD25^+^Foxp3^+^) in tumor tissue of 4T1 tumor-bearing mice.

**
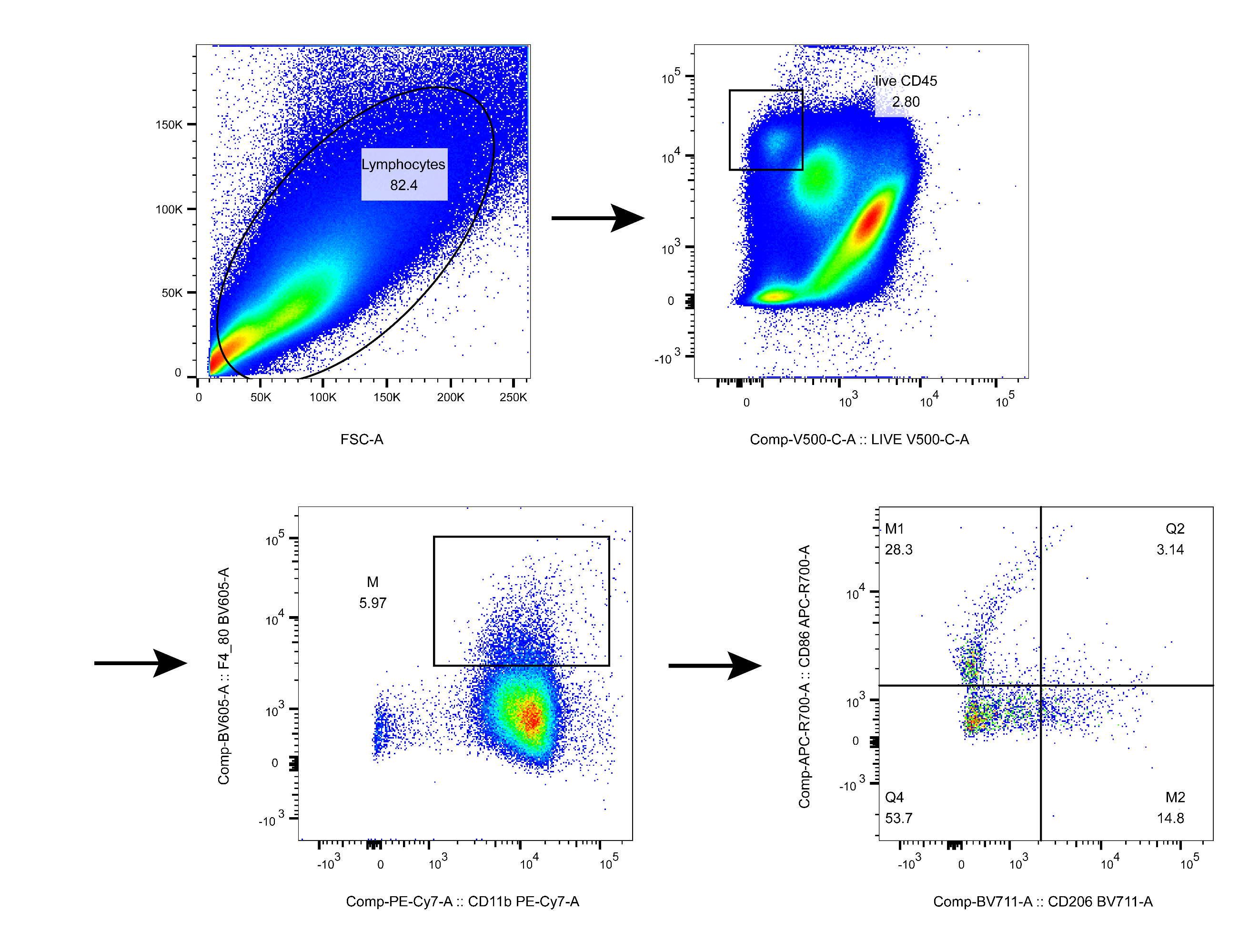
**

**Figure S22.** Representative flow cytometry gating strategies for M1 (CD11b^+^F4/80^+^CD86^+^) and M2 (CD11b^+^F4/80^+^CD206^+^) macrophages in tumor tissue of 4T1 tumor-bearing mice.

**Table S1.** Particle size and ζ-potential of PBAE-S-AZA/pMax-GFP polyplexes.

| Sample | Mass ratio | Diameter (nm) | ζ-potential |
| --- | --- | --- | --- |
| PBAE-S-AZA/pMax-GFP  polyplexes | 10:1 | 198.98±2.06 | 4.52±1.49 |
|  | 25:1 | 169.81±1.41 | 4.56±1.82 |
|  | 50:1 | 102.76±0.08 | 10.87±0.95 |
|  | 75:1 | 150.18±1.34 | 13.86±0.32 |
|  | 100:1 | 269.48±26.74 | 13.93±0.56 |

**Table S2.** The sequences of sgRNA oligos used for CRISPR/dCas9-KRAB system.

| sgPD-L1 | Sequence (5’ to 3’) |
| --- | --- |
| sgPD-L1-1 S | CACCGGAGATAAGACCAGGAAATCG |
| sgPD-L1-1 AS | AAACCGATTTCCTGGTCTTATCTCC |
| sgPD-L1-2 S | CACCGGGCTTCGGTTTCACAGACAG |
| sgPD-L1-2 AS | AAACCTGTCTGTGAAACCGAAGCCC |
| sgPD-L1-3 S | CACCGTTATCTCATAGTATTTCCTG |
| sgPD-L1-3 AS | AAACCAGGAAATACTATGAGATAAC |
| sgPD-L1-4 S | CACCGAACGAAACTAGGCTTCGGTG |
| sgPD-L1-4 AS | AAACCACCGAAGCCTAGTTTCGTTC |
| sgPD-L1-5 S | CACCGACAACCGGGCTGCTACTGAG |
| sgPD-L1-5 AS | AAACCTCAGTAGCAGCCCGGTTGTC |
